# Whole-genome Sequencing Reveals Autooctoploidy in Chinese Sturgeon and Its Evolutionary Trajectories

**DOI:** 10.1093/gpbjnl/qzad002

**Published:** 2023-12-13

**Authors:** Binzhong Wang, Bin Wu, Xueqing Liu, Yacheng Hu, Yao Ming, Mingzhou Bai, Juanjuan Liu, Kan Xiao, Qingkai Zeng, Jing Yang, Hongqi Wang, Baifu Guo, Chun Tan, Zixuan Hu, Xun Zhao, Yanhong Li, Zhen Yue, Junpu Mei, Wei Jiang, Yuanjin Yang, Zhiyuan Li, Yong Gao, Lei Chen, Jianbo Jian, Hejun Du

**Affiliations:** Hubei Key Laboratory of Three Gorges Project for Conservation of Fishes, Yichang 443100, China; Chinese Sturgeon Research Institute, China Three Gorges Corporation, Yichang 443100, China; Yangtze River Biodiversity Research Center, China Three Gorges Corporation, Wuhan 430014, China; BGI-Shenzhen, Shenzhen 518083, China; BGI Genomics, BGI-Shenzhen, Shenzhen 518083, China; Hubei Key Laboratory of Three Gorges Project for Conservation of Fishes, Yichang 443100, China; Chinese Sturgeon Research Institute, China Three Gorges Corporation, Yichang 443100, China; Yangtze River Biodiversity Research Center, China Three Gorges Corporation, Wuhan 430014, China; Hubei Key Laboratory of Three Gorges Project for Conservation of Fishes, Yichang 443100, China; Chinese Sturgeon Research Institute, China Three Gorges Corporation, Yichang 443100, China; Yangtze River Biodiversity Research Center, China Three Gorges Corporation, Wuhan 430014, China; BGI Genomics, BGI-Shenzhen, Shenzhen 518083, China; BGI Genomics, BGI-Shenzhen, Shenzhen 518083, China; Department of Biotechnology and Biomedicine, Technical University of Denmark, Lyngby 2800, Denmark; Hubei Key Laboratory of Three Gorges Project for Conservation of Fishes, Yichang 443100, China; Chinese Sturgeon Research Institute, China Three Gorges Corporation, Yichang 443100, China; Yangtze River Biodiversity Research Center, China Three Gorges Corporation, Wuhan 430014, China; Hubei Key Laboratory of Three Gorges Project for Conservation of Fishes, Yichang 443100, China; Chinese Sturgeon Research Institute, China Three Gorges Corporation, Yichang 443100, China; Yangtze River Biodiversity Research Center, China Three Gorges Corporation, Wuhan 430014, China; River Basin Complex Administration Center, China Three Gorges Corporation, Yichang 443100, China; Hubei Key Laboratory of Three Gorges Project for Conservation of Fishes, Yichang 443100, China; Chinese Sturgeon Research Institute, China Three Gorges Corporation, Yichang 443100, China; Yangtze River Biodiversity Research Center, China Three Gorges Corporation, Wuhan 430014, China; BGI Genomics, BGI-Shenzhen, Shenzhen 518083, China; Hubei Key Laboratory of Three Gorges Project for Conservation of Fishes, Yichang 443100, China; Chinese Sturgeon Research Institute, China Three Gorges Corporation, Yichang 443100, China; Yangtze River Biodiversity Research Center, China Three Gorges Corporation, Wuhan 430014, China; Hubei Key Laboratory of Three Gorges Project for Conservation of Fishes, Yichang 443100, China; Chinese Sturgeon Research Institute, China Three Gorges Corporation, Yichang 443100, China; Yangtze River Biodiversity Research Center, China Three Gorges Corporation, Wuhan 430014, China; Hubei Key Laboratory of Three Gorges Project for Conservation of Fishes, Yichang 443100, China; Chinese Sturgeon Research Institute, China Three Gorges Corporation, Yichang 443100, China; Yangtze River Biodiversity Research Center, China Three Gorges Corporation, Wuhan 430014, China; Hubei Key Laboratory of Three Gorges Project for Conservation of Fishes, Yichang 443100, China; Chinese Sturgeon Research Institute, China Three Gorges Corporation, Yichang 443100, China; Yangtze River Biodiversity Research Center, China Three Gorges Corporation, Wuhan 430014, China; BGI Genomics, BGI-Shenzhen, Shenzhen 518083, China; BGI-Sanya, BGI-Shenzhen, Sanya 572025, China; BGI-Shenzhen, Shenzhen 518083, China; BGI-Sanya, BGI-Shenzhen, Sanya 572025, China; Hubei Key Laboratory of Three Gorges Project for Conservation of Fishes, Yichang 443100, China; Chinese Sturgeon Research Institute, China Three Gorges Corporation, Yichang 443100, China; Yangtze River Biodiversity Research Center, China Three Gorges Corporation, Wuhan 430014, China; Hubei Key Laboratory of Three Gorges Project for Conservation of Fishes, Yichang 443100, China; Chinese Sturgeon Research Institute, China Three Gorges Corporation, Yichang 443100, China; Yangtze River Biodiversity Research Center, China Three Gorges Corporation, Wuhan 430014, China; Hubei Key Laboratory of Three Gorges Project for Conservation of Fishes, Yichang 443100, China; Chinese Sturgeon Research Institute, China Three Gorges Corporation, Yichang 443100, China; Yangtze River Biodiversity Research Center, China Three Gorges Corporation, Wuhan 430014, China; Yangtze Eco-Environment Engineering Research Center, China Three Gorges Corporation, Beijing 100038, China; Hubei Key Laboratory of Three Gorges Project for Conservation of Fishes, Yichang 443100, China; Chinese Sturgeon Research Institute, China Three Gorges Corporation, Yichang 443100, China; River Basin Complex Administration Center, China Three Gorges Corporation, Yichang 443100, China; BGI Genomics, BGI-Shenzhen, Shenzhen 518083, China; Department of Biotechnology and Biomedicine, Technical University of Denmark, Lyngby 2800, Denmark; Hubei Key Laboratory of Three Gorges Project for Conservation of Fishes, Yichang 443100, China; Chinese Sturgeon Research Institute, China Three Gorges Corporation, Yichang 443100, China; Yangtze River Biodiversity Research Center, China Three Gorges Corporation, Wuhan 430014, China

**Keywords:** Chinese sturgeon, Whole-genome sequencing, Autooctoploid, Polyploidization and diploidization, Whole-genome duplication

## Abstract

The order Acipenseriformes, which includes sturgeons and paddlefishes, represents “living fossils” with complex genomes that are good models for understanding whole-genome duplication (WGD) and ploidy evolution in fishes. Here, we sequenced and assembled the first high-quality chromosome-level genome for the complex octoploid *Acipenser sinensis* (Chinese sturgeon), a critically endangered species that also represents a poorly understood ploidy group in Acipenseriformes. Our results show that *A*. *sinensis* is a complex autooctoploid species containing four kinds of octovalents (8*n*), a hexavalent (6*n*), two tetravalents (4*n*), and a divalent (2*n*). An analysis taking into account delayed rediploidization reveals that the octoploid genome composition of Chinese sturgeon results from two rounds of homologous WGDs, and further provides insights into the timing of its ploidy evolution. This study provides the first octoploid genome resource of Acipenseriformes for understanding ploidy compositions and evolutionary trajectories of polyploid fishes.

## Introduction

The order Acipenseriformes, which includes sturgeons and paddlefishes, is an ancient group of fishes with a wide distribution in the Northern Hemisphere. Many species of Acipenseriformes are threatened or endangered, particularly due to their commercial values for meat and caviar. As “living fossils”, Acipenseriformes species retain primitive characteristics (such as a heterocercal tail and cartilaginous skeleton) and occupy the basal position of Actinopterygii phylogeny [1]. They also exhibit a slow rate of evolution [2], complex genomes (half of their chromosomes are micro-chromosomes), and complex ploidy, with the order divisible into three ploidy classifications: Group A (∼ 120 chromosomes with nuclear DNA content of 3.2–4.6 pg), Group B (∼ 240 chromosomes with nuclear DNA content of 6.1–9.6 pg), and Group C (∼ 360 chromosomes with nuclear DNA content of 13.1–14.2 pg) [3,4]. A better understanding of Acipenseriformes could aid in conservation efforts and provide insights into the understanding of whole-genome duplication (WGD) and ploidy evolution in fishes [5–7].

WGDs are very common in the evolution of fishes [8–10], and subsequent rediploidization further increases the complexity of the genomes. Both Acipenseriformes and teleosts (ray-finned fishes except primitive bichirs, sturgeons, paddlefishes, freshwater garfishes, and bowfins) have undergone at least three rounds of WGDs. The first two rounds include the first-round WGD (1R) that occurred ∼ 600 million years ago (MYA) and a jawed vertebrate-specific second-round WGD (2R) that occurred after the divergence of lamprey and jawed vertebrates. Teleosts then underwent a teleost-specific third-round WGD (Ts3R) [11–14], whereas Acipenseriformes underwent an independent Acipenseriformes-specific third-round WGD (As3R) [11]. Acipenseriformes species are thought to have undergone a delayed rediploidization, in which a species radiates an extensive time after a WGD event (*i.e.*, a timescale on the order of millions of years) [15], resulting in complex ploidies. However, the ploidy compositions of most Acipenseriformes species have been challenging to clarify. The debates regarding ploidy, for example, whether Groups A and B are diploid and tetraploid [5] or are instead tetraploid [16] and octoploid [17–19], or even are paleotetraploidy *versus* modern/functional diploidy in the case of Group A [4], have lasted for half a century [20,21]. It has been difficult to end the debate solely by relying on traditional DNA content measurements, cytogenetics, and molecular biology techniques [18,22–24], and thus whole-genome sequencing (WGS) analyses are needed to help resolve these outstanding questions. Moreover, efforts in the estimation of polyploidization and speciation time need to take into account rediploidization effects appropriately.

The “lineage-specific ohnologue resolution” (LORe) model (Figure S1A) was proposed to address delayed rediploidization in sister lineages that share the common WGD [15]. In the LORe model, speciation precedes rediploidization, allowing for independent ohnologue divergence in sister lineages that share an ancestral WGD event. A phylogenetic implication of LORe is the absence of 1:1 orthology between ohnologue pairs from different lineages, leading to the definition of the term “tetralog” to describe a 2:2 homology relationship between ohnologues in sister lineages. Order differences in divergency and polyploidization, as well as the influence of species characteristics, will lead to differential enrichment of LORe and “ancestral ohnologue resolution” (AORe) [15,25]. Thus, the estimation of polyploidization and speciation time that ignores the effects of LORe and AORe would produce an inaccurate result based on the traditional methods of globally homologous comparison. Previous reports on WGD and rediploidization processes only focused on Acipenseriformes species of Group A but not on species of Group B mainly because the Group B genomic resources were lacking and the influence of delayed rediploidization on the analyses was not considered [11,26], although the phylogenetic analysis of autopolyploid American paddlefish genome studies based on traditional methods of globally homologous comparison did suggest an influence of delayed rediploidization [26].

To address the lack of Group B resources and to further clarify ploidy in Acipenseriformes species, we examined the Group B Acipenseriformes species *Acipenser sinensis* (Chinese sturgeon) by WGS. *A*. *sinensis* is a critically endangered large fish in China and also a sturgeon species of world-wide concerned with the lowest distribution latitude [27]. *A*. *sinensis* has ∼ 264 chromosomes, including 124 macro-chromosomes and ∼ 140 micro-chromosomes [18,28], and is also considered paleooctoploid [29]. Based on our WGS and subsequent analysis, we presented the first high-quality genome assembly of *A*. *sinensis* and its ploidy composition post-rediploidization. Furthermore, by combining the genomic data of *A*. *sinensis* (Pacific group of Acipenseridae, Group B), *Acipenser ruthenus* (Pacific group of Acipenseridae, Group A) [30], and *Polyodon spathula* (Polyodontidae, Group A) [26] and integrating assessment of the influence of the delayed rediploidization, we also uncovered the evolutionary trajectories of *A*. *sinensis*.

## Results

### *A*. *sinensis* genome sequencing, assembly, and annotation

**Figure 1:**
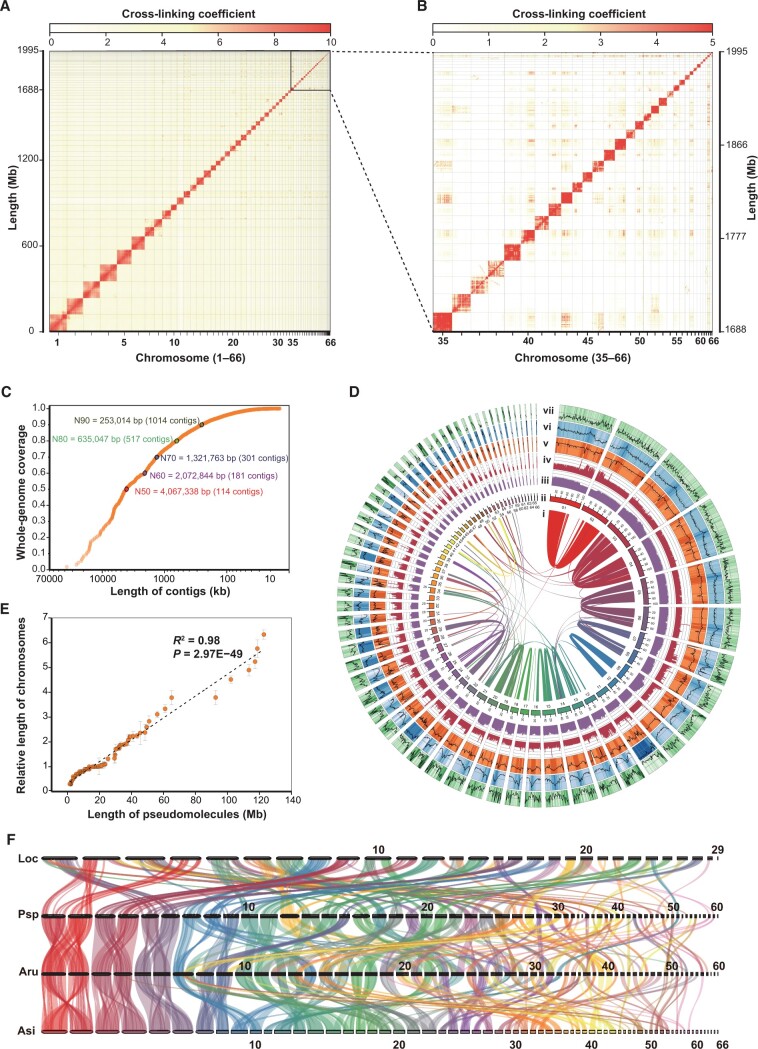
Genome assembly and evaluation of *Acipenser sinensis* **A**. Heatmap of interactions within and among chromosomes based on Hi-C data. Each dark red block with a high cross-linking coefficient and clear boundaries is a chromosome region. A total of 66 chromosomes were segmented. **B**. Mosaics of 33 detailed micro-chromosomes (chromosome Nos. 35–66) were displayed. **C**. Length distribution of contigs in the whole-genome assembly. **D**. Features of 66 assembled chromosomes. Tracks from the inner to the outside are indicated as follows: (i) relationships between collinearity blocks; (ii) pseudo-chromosomes; (iii) frequencies of suballeles (0–0.5); (iv) sequencing depths (0–200×); (v) GC contents (20%–50%); (vi) TE contents (0%–100%); and (vii) gene contents (0–50). **E**. Correlation analysis between pseudomolecule length and relative length of chromosomes. **F**. Relationships of collinearity blocks among Loc, Psp, Aru, and Asi. Loc, *Lepisosteus oculatus*; Psp, *Polyodon spathula*; Aru, *Acipenser ruthenus*; Asi, *Acipenser sinensis*; Hi-C, high-through chromosome conformation capture; TE, transposable element.

To obtain a high-quality genome assembly, DNA from a meiotic gynogenetic male *A*. *sinensis* was sequenced by the combination of the Illumina platform, the PacBio platform, and high-through chromosome conformation capture (Hi-C) sequencing technology. We obtained 421.58-Gb clean Illumina short-read data (Table S1), 221.96-Gb clean PacBio long-read data (Table S2), and 172.87-Gb clean Hi-C data (Table S3). Illumina and PacBio reads were assembled into the initial contigs of ∼ 1.99 Gb with an N50 size of ∼ 4.07 Mb (**Table 1**). Clean Hi-C reads were applied to anchor contigs into 66 scaffolds corresponding to 66 chromosomes of two monoploid genomes (**Figure 1**A and B; Table 1). The final genome assembly was 1.99 Gb with a scaffold N50 size of ∼ 48.46 Mb, and 98.3% of assembled sequences were assigned to chromosomes (Figure 1C and D; Table 1). This genome assembly size was comparable to the size estimated by a *k*-mer-based method (1.975 Gb) and one-quarter of the DNA content (2.27 pg/2C) estimated by the flow cytometer (Figures S2 and S3; Table S4). We observed a high correlation (*R*^2^ = 0.98; *P* = 2.97E−49) between the sizes of 66 assembled chromosomes and the relative physical length of chromosomes based on our karyotype results (Figure 1E; Table S5). The completeness of genome assembly was 95.6%, including 60.0% “complete and single-copy Benchmarking Universal Single-Copy Orthologs (BUSCOs)” and 35.6% “complete and duplicated BUSCOs” (Table S6). Using the BWA alignment results with 500-bp data, we further determined that the mapping rate and coverage of the sequences were 99.67% and 93.62%, respectively.

**Table 1:** Statistics of genome assembly of three sequenced Acipenseriformes species

Parameter	*Acipenser sinensis*	***Acipenser ruthenus*** [11]	***Polyodon spathula*** [26]
Ploidy	8*n*	4*n*	4*n*
Assembly size (bp)	1,995,374,126	1,830,501,248	1,542,083,420
Contig N50 (bp)	4,067,338	600,839	3,441,286
Scaffold N50 (bp)	48,462,029	42,361,471	48,906,729
Contig N90 (bp)	253,014	72,599	255,888
Scaffold N90 (bp)	13,550,145	3,341,419	7,546,224
Length of gaps (bp)	1,560,502	1,254,212	1,696,466
GC content	40.76%	39.76%	39.02%
No. of chromosomes	66	60	60
Chromosome length (bp)	1,962,255,781	1,651,776,498	1,487,634,427
Chromosome anchoring rate	98.3%	90.2%	96.5%
Complete BUSCOs of assembly	95.60%	98.30%	93.7%
No. of coding genes	36,837	35,379	30,260
Complete BUSCOs of genes	93.10%	NA	NA

*Note*: BUSCOs, Benchmarking Universal Single-Copy Orthologs; NA, not available.

We integrated *de novo*, protein homology-based, and RNA sequencing (RNA-seq) data-assisted methods to predict gene structures (Table S7). We predicted a total of 36,837 protein-coding genes, with an average length of 14.3 kb (**Table 2**). BUSCO evaluation indicated that the annotation covered 93.1% of the vertebrate core gene sets (Table S8). We annotated a total of 34,114 (92.61%) functional genes among the predicted protein-coding genes, with 91.78%, 82.36%, 78.18%, 69.09%, 91.18%, 84.21%, and 57.81% of the predicted protein-coding genes matching Non-Redundant Protein Sequence Database (NR) in National Center of Biotechnology Information (NCBI), Swiss-Prot, Kyoto Encyclopedia of Genes and Genomes (KEGG), EuKaryotic Orthologous Groups (KOG), Translation of European Molecular Biology Laboratory (TrEMBL), InterPro, and Gene Ontology (GO) databases, respectively (Figure S4; Table S9). Meanwhile, we identified 991.35-Mb repetitive elements (49.68% of the *A. sinensis* genome), containing 204.28-Mb tandem repeats (10.24% of the genome) (Table S10) and 903.50-Mb transposable elements (TEs) (45.28% of the genome). Among the assembled genome, 16.34% are long interspersed nuclear elements (LINEs), 17.43% are long terminal repeats (LTRs), 1.88% are short interspersed nuclear elements (SINEs), and 17.67% are class II TEs (DNA transposons) (Table S11). Overall, we sequenced and assembled a high-quality genome of *A*. *sinensis*.

**Table 2:** General statistics of predicted protein-coding genes

Method	Gene set	No. of predicted protein-coding genes	Average gene length (bp)	Average CDS length (bp)	Average exon count per gene	Average exon length (bp)	Average intron length (bp)
*De novo* prediction	AUGUSTUS	105,618	10,958.13	1,231.69	4.93	249.99	2476.87
	SNAP	132,324	14,471.77	1,495.38	5.95	251.45	2623.12
Protein homology-based prediction	*Callorhinchus milii*	88,902	6333.78	923.12	3.70	249.72	2006.53
	*Danio rerio*	94,544	6991.85	1051.28	4.06	259.05	1942.49
	*Latimeria chalumnae*	140,860	4618.43	876.97	3.13	280.21	1756.83
	*Lepisosteus oculatus*	102,019	7265.95	1007.46	4.15	242.63	1985.44
	*Petromyzon marinus*	94,541	3151.53	762.17	2.37	322.15	1749.33
RNA-seq-based prediction	RNA	23,727	6467.33	1097.87	6.42	170.93	1092.26
Total		36,837	14,306.64	1392.56	8.03	173.37	1708.37

*Note* **:** The average transcript length did not contain UTR. Results of *de novo* prediction and protein homology-based prediction were consolidated using the program GLEAN. UTR, untranslated region; CDS, coding sequence; RNA-seq, RNA sequencing.

### High-quality *A*. *sinensis* genome assembly supported by collinearity analysis and phylogenetic tree construction

To analyze the genome assembly quality and chromosome evolution, we performed a collinearity analysis among the genomes of three sequenced Acipenseriformes species (*P. spathula*, *A. ruthenus*, and *A. sinensis*) and a closely related species of Acipenseriformes (*Lepisosteus oculatus*) on the evolutionary tree. A total of 25,687 collinear genes were detected in the *A*. *sinensis* internal genome. We also obtained collinear genes between *A*. *sinensis* and *A*. *ruthenus* (31,348 genes), *A*. *sinensis* and *P*. *spathula* (28,945 genes), *A*. *sinensis* and *L*. *oculatus* (16,062 genes), *A*. *ruthenus* and *P*. *spathula* (34,031 genes), and *P*. *spathula* and *L*. *oculatus* (18,053 genes) (Table S12). The six relatively large chromosomes displayed the definite two-to-two collinearity relationship between *A. sinensis*, *A. ruthenus*, and *P. spathula* (Figure 1F).

Meanwhile, 21,410 gene families were obtained by clustering homologous gene sequences among 13 species. We chose these fish species for phylogenetic analysis based on three principles: (1) they have been whole-genome sequenced, (2) they represent important branches or nodes within the phylogenetic tree, and (3) they are typical representative species or model species. According to these principles, *Petromyzon marinus* represents jawless fishes, *Callorhinchus milii* represents cartilaginous fishes, *Latimeria chalumnae* represents ancient fishes and lobe-finned fishes, *Polypterus senegalus* represents polypterids, *L. oculatus* represents the evolution node species of Actinopterygian and bony fishes, *Salmo salar* represents a highly tetraploid species, *Cyprinus carpio* represents typical allopolyploid fishes, *Gadus morhua* represents typical bony fishes, and *P*. *spathula* represents paddlefish. *A. ruthenus* represents the Group A population of sturgeon in the Atlantic branch of the sturgeon family (with a chromosome number of ∼ 120), and *A. sinensis* represents the Group B species of sturgeon in the Pacific branch of the sturgeon family (with a chromosome number of ∼ 240). Lastly, *Oryzias latipes* and *Danio rerio* were included as important model organism species.

We constructed the phylogenetic tree with 2096 genes using two methods, PhyML [31] (Table S13) and ASTRAL [32] (Figure S5), both of which yielded a phylogenetic tree with the same topological structure. The resulting trees revealed that *A. sinensis* and *A. ruthenus* have the same Acipenseridae ancestor, whereas *P. spathula* is a sister lineage with the Acipenseridae and is classified as Polyodontidae. The trees also showed that as a cluster of representative ancient species, the Acipenseriformes and Lepisosteiformes diverged from the same evolutionary branch (Figure S6). The results are consistent with the previous study [33] and thus together with the collinearity analysis, verify the high assembly quality and integrity of our genomic dataset and analysis.

### Octoploid features of *A. sinensis* indicated by karyotype, simple sequence repeat, and single nucleotide polymorphism analyses

Traditional karyotype analysis showed that *A*. *sinensis* has 264 chromosomes (Figure S7), approximately four times the 60-chromosome karyotype of the common diploid ancestor of Acipenseriformes [18,28,34]. Thus, *A*. *sinensis* was presumed to be an octoploid species. To further explore *A*. *sinensis* ploidy at the genome level, we obtained 200-Gb genome sequences of a normal reproductive animal, instead of a meiotic gynogenetic animal, using the BGISEQ platform. We analyzed genome-wide simple sequence repeats (SSRs) and single nucleotide polymorphisms (SNPs) based on the data. SSR analysis showed that the largest number of alleles at a single locus was up to eight (Figure S8; Table S14), implying that the species has eight homologous chromosomes.

**Figure 2:**
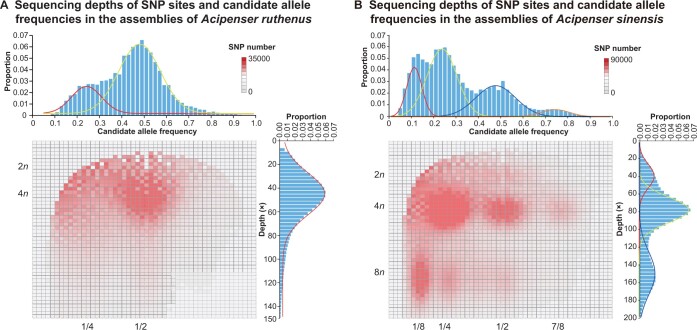
Sequencing depths and allele frequencies Sequencing depths of SNP sites and allele frequencies in the assemblies of *Acipenser ruthenus* (**A**) and *Acipenser sinensis* (**B**). In the main plot, the X and Y axes represent allele frequency and SNP site sequencing depth, respectively. The right and upper bar charts show the number of hits at the given frequency and depth. SNP, single nucleotide polymorphism.

For comparative analysis of ploidy, we further identified 22,324,005 and 9,826,321 SNPs in *A*. *sinensis* and *A*. *ruthenus*, respectively, and the heterozygosity of *A*. *sinensis* (1.12%) was approximately two folds that of the tetraploid *A*. *ruthenus* (0.54%) (Table S15). We evaluated and plotted the candidate allele frequency and ploidy (*n*) of the SNP sites. In *A*. *ruthenus*, most of the candidate allele frequencies of SNP sites displayed 1/2 and 1/4, but both depths pointed to 4*n* (∼ 46×) (**Figure 2**A), indicating that the genotypes are AABB and AAAB. This implies that *A*. *ruthenus* experienced an autotetraploidization event followed by a rediploidization event, similar to the conclusion drawn in a previous study by Du and his colleagues [11]. *A*. *sinensis* has more complex ploidy, and we observed the presence of bivalents (2*n*), tetravalents (4*n*), and octovalents (8*n*) in the analysis (Figure 2B). *A*. *sinensis* exhibited four peaks in the SNP frequency curve, whereas *A*. *ruthenus*, the tetraploid sturgeon, only exhibited two peaks. The main peak of ploidy in *A*. *sinensis* was 4*n*, pointing to 1/4 and 1/2 candidate allele frequencies. Most importantly, we detected the first peak at the position of 1/8 candidate allele frequency in *A. sinensis*. This peak mostly pointed to 8*n* ploidy, which suggests that the eight monoploids have high similarity and reveals the octoploid features of *A. sinensis*.

### Autoocotoploid features of *A*. *sinensis* revealed by ploidy composition and TE analyses

**Figure 3:**
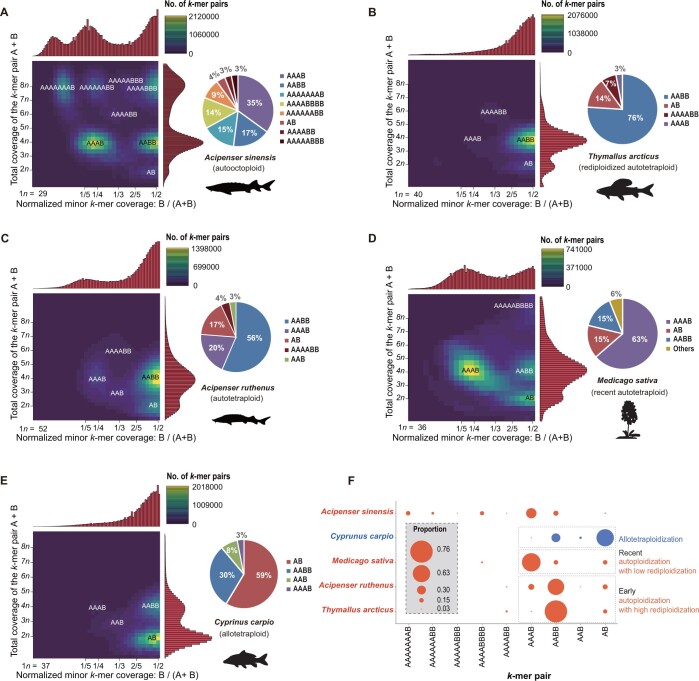
Ploidy compositions of five polyploid species **A**. Ploidy composition of *Acipenser sinensis*. **B**. Ploidy composition of *Thymallus arcticus*. **C**. Ploidy composition of *Acipenser ruthenus*. **D**. Ploidy composition of *Medicago sativa*. **E**. Ploidy composition of *Cyprinus carpio*. The letters A and B in the Smudgeplot of the five polyploid species represent a pair of heterozygous *k*-mers with only one SNP difference. The brightness of each smudge is determined by the number of heterozygous *k*-mer pairs that fall within it. The percentage of each genotype is presented in the pie chart on the right. **F**. The distribution of nine genotypes (*k*-mers) in the five species. The main compositions of tetraploid species are framed by dashed lines. AB accounts for a higher proportion in allotetraploid, while AAAB and AABB are higher proportions in autotetraploid. With increasing rediploidization, the proportion of AAAB decreases while the proportion of AABB increases.

To further assess the ploidy composition of *A*. *sinensis*, we performed Smudgeplot analysis based on the depth and frequency of heterozygous *k*-mer pairs (with only 1-nt difference, presented as A and B symbols) using the BGISEQ data from the normal reproductive animal. The results reveal that the ploidy composition of *A*. *sinensis* is extremely complex, containing 41% of four octovalent (8*n*) *k*-mers (AAAAAAAB, AAAAAABB, AAAAABBB, and AAAABBBB), 3% of a hexavalent (6*n*) *k*-mer (AAAABB), 52% of two tetravalent (4*n*) *k*-mers (AAAB and AABB), and 4% of a divalent (2*n*) *k*-mer (AB) (**Figure 3**A). The high proportion of octovalent *k*-mers demonstrates that the *A*. *sinensis* genome has typical octoploid characteristics. Moreover, we compared the results of this analysis for *A*. *sinensis* with those for four representative and well-studied tetraploid species — *Thymallus arcticus* (rediploidized autotetraploid) [35], *A. ruthenus* (rediploidized autotetraploid) [11], *Medicago sativa* (recently duplicated autotetraploid) [36], and *C. carpio* (allotetraploid) [37,38] — using the Smudgeplot analysis. *T. arcticus* and *A. ruthenus*, both rediploidized autotetraploids, showed common characteristics whereby AABB accounted for the dominant proportion and a lower and nearly equivalent proportion of AB. The proportion of AAAB in *T*. *arcticus* (3%) (Figure 3B) was much lower than that in *A. ruthenus* (20%) (Figure 3C), which suggests that *T. arcticus* has a higher extent of rediploidization than *A. ruthenus* due to the lower evolution rate of Acipenseriformes, consistent with previous research [11]. In *M. sativa*, AAAB accounted for the dominant proportion (63%), whereas AABB only accounted for 15% (Figure 3D), suggesting the more recent WGD of *M. sativa* (∼ 58 MYA) [36] in comparison to *T. arcticus* (80–100 MYA)[35] and *A. ruthenus* (∼ 180 MYA) [11] that resulted in a higher homology and lower rediploidization level. It is well known that *C. carpio* is one of the representative allotetraploid teleosts. Two ancient progenitor species (AA and BB) of *C. carpio* diverged 23 MYA, and they independently survived to subsequently produce the current allotetraploid *C. carpio* (AABB) by hybridization ∼ 12.3 MYA [37,38]. Ploidy analysis of *C. carpio* showed that AB, AABB, and AAAB *k*-mers accounted for 59%, 30%, and 3%, respectively (Figure 3E). The distribution of heterozygous *k*-mers in the four tetraploid species showed that AB was the dominant proportion in allotetraploid species (*C. carpio*). This was due to the recombination of significantly differential subgenomes which led to *k*-mer pair sequences mispairing to tetravalents. Our results suggest that AAAB accounts for the dominant composition in the autotetraploid species with lower rediploidization (*M*. *sativa*), whereas AABB is the dominant composition in the autotetraploid species with high rediploidization (*T*. *arcticus* and *A*. *ruthenus*) (Figure 3F). Compared with the tetraploid *A*. *ruthenus*, the octoploid *A*. *sinensis* may have undergone an additional round of WGD, resulting in the overlap of the genotypes we observed. Furthermore, the proportion of autopolyploidization characteristics, including AAAAAAAB, AAAAAABB, AAAAABBB, and AAAB, was up to 62% in *A*. *sinensis*, suggesting that the species is an autooctoploid species.

Some repeats may be expanded specifically at each progenitor of subgenomes due to independent evolution before allotetraploid hybrids. Thus, the burst of distinctive TEs might be closely related to separating from two subgenomes in the allopolyploid genomes [39–42]. The burst of distinctive TEs might be closely related to the separate evolution of ancestors from two subgenomes in the allopolyploid genomes [39–42]. Here, we attempted to identify the distinctive TEs between homologous sequences to identify the auto- or allo-polyploidization process of *A*. *sinensis*. However, we did not observe significantly specific TEs (Figure S9; Tables S16 and S17), which excluded the possibility of allooctoploidy, similar to what has been concluded for *A. ruthenus* [11]. These results imply that *A. sinensis* has undergone homologous duplications, indicating that it is an autooctoploid species.

### A common WGD and the speciation time revealed by LORe and AORe analyses

**Figure 4:**
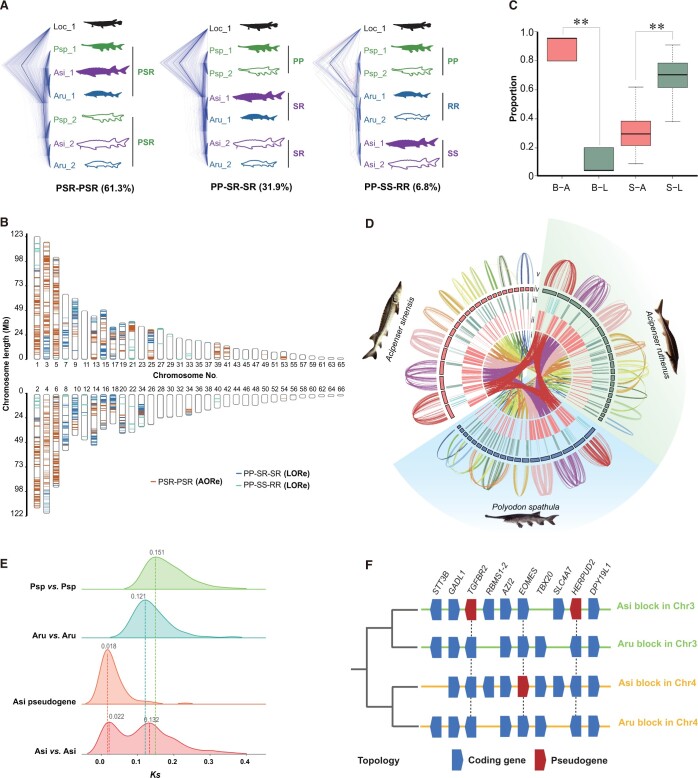
WGD events and divergence of Acipenseriformes based on LORe and AORe analyses **A**. Main topological structures based on different assumptions. **B**. Distributions of AORe and LORe on chromosomes. The red, blue, and green lines represent AORe in PSR-PSR, LORe in PP-SR-SR, and LORe in PP-SS-RR, respectively. **C**. Distributions of AORe and LORe homology on macro- and micro-chromosomes. B–A indicates the ratio of AORe on macro-chromosomes; B–L indicates the ratio of LORe on macro-chromosomes; S–A indicates the ratio of AORe on micro-chromosomes; S–L indicates the ratio of LORe on micro-chromosomes. **, *P* < 0.01 (Student’s *t*-test). **D**. Distributions of AORe and LORe in the genomes of *Acipenser sinensis*, *Acipenser ruthenus*, and *Polyodon spathula*. Tracks from the inner to the outside correspond to (i) collinearity among *Acipenser sinensis*, *Acipenser ruthenus*, and *Polyodon spathula*, (ii) locations of AORe genes, (iii) locations of LORe genes, (iv) chromosomes with more than 20 genes (red, green, and blue blocks represent chromosomes of *Acipenser sinensis*, *Acipenser ruthenus*, and *Polyodon spathula*, respectively), and (v) collinearity within each species. **E**. Estimation of WGD and divergence events occurring among *Acipenser sinensis*, *Acipenser ruthenus*, and *Polyodon spathula* by *Ks*. **F**. An example of coding genes and pseudogenes within one block in subgenome-like of *Acipenser sinensis* and *Acipenser ruthenus*. WGD, whole-genome duplication; LORe, lineage-specific ohnologue resolution; AORe, ancestral ohnologue resolution; P, *Polyodon spathula*; S, *Acipenser sinensis*; R, *Acipenser ruthenus*; *Ks*, synonymous substitution rate.

To uncover a more accurate WGD and divergence time for *A*. *sinensis* based on LORe and AORe, we screened 1438 gene families with collinearity among the genomes of *A*. *sinensis* (S), *A*. *ruthenus* (R), *P*. *spathula* (P), and *L. oculatus* with the gene copy number of 2:2:2:1. We constructed 1438 topologies using the screened gene families (Table S18), and three types of representative topologies that accurately represented the AORe model (PSR-PSR for topology name) and the LORe model (PP-SR-SR and PP-SS-RR for topology names) (**Figure 4**A, Figure S1B) were collected (736 gene families). The PSR-PSR type was the dominant topology and accounted for 61.3% of 736 screened gene families, followed by PP-SR-SR (31.9%) and PP-SS-RR (6.8%). The PSR-PSR type was the most abundant topology, indicating that the three species underwent a common duplication event, as otherwise this observation cannot be explained (Figure S1B). In addition, the PP-SR-SR ratio was the highest (31.9%), implying that the two families of Acipenseriformes diverged after As3R but before complete rediploidization. Thus, LORe occurred during the evolution of Acipenseriformes species (Figure S1B). The low percentage of PP-SS-RR suggests that there was a nearly complete rediploidization event in the ancestors of *A*. *sinensis* and *A*. *ruthenus* before their divergence and speciation (Figure S1B). Allotetraploids are expected to show disomic inheritance (genetic diploidy) as soon as they are formed and the rediploidization is immediately completed [42]. As a result, high levels of LORe only appeared in autopolyploidy. Our results imply that the Acipenseriformes species share a common polyploidization process. In addition, the distribution of LORe and AORe on the *A*. *sinensis*, *A*. *ruthenus*, and *P*. *spathula* chromosomes showed that AORe was mainly distributed on the 1–6 macro-chromosomes, whereas LORe tended to occur on medium- and micro-chromosomes, probably due to their instability (Figure 4B–D; Table S19). The synonymous substitution rates (*Ks*) were larger on the macro-chromosomes than those on the medium- and micro-chromosomes (Figure S10). This supported the distribution features of AORe and LORe on the chromosomes.

The *Ks* values in coding genes and unitary pseudogenes were calculated for estimating the time of the *A*. *sinensis*-specific WGD (Ass4R) (Figure S11). Using As3R [time (T) = 210.7 MYA; *Ks* = 0.132] as a reference, Ass4R (*Ks* = 0.022) was calculated at ∼ 35.12 MYA (Figure 4E). Furthermore, referring to a previous study [42], we speculated that some missing homoeologues, which were not detected as coding genes, would be presented in the *A*. *sinensis* genome as new unitary pseudogenes following Ass4R. We screened out 344 pseudogenes with high assurance from the gene families (with quadrivalent pairing collinearity in *A*. *ruthenus* and *A*. *sinensis* and each gene family conforming to the AORe model) (Figure 4F, Figure S12). Based on the accumulation of more nonsynonymous substitution rates (*Ka*) than expected [42,43], we estimated that most of these pseudogenes escaped evolutionary constraint ∼ 28.7 MYA (Figure 4E), which is in line with the expectation that they occurred shortly after Ass4R. Based on calculated *Ks* values of three intra-species in Acipenseriformes (*A*. *sinensis*: *Ks* = 0.132; *A*. *ruthenus*: *Ks* = 0.121; *P*. *spathula*: *Ks* = 0.151) with common WGD time (T = 210.7 MYA), the absolute substitution rates of the sturgeons were 3.13 × 10^−10^, 2.87 × 10^−10^, and 3.58 × 10^−10^ per year, respectively, as calculated by the formula: *Ks*/(2T). *P*. *spathula* has the highest absolute substitution rate, followed by *A*. *sinensis* and *A*. *ruthenus*.

**Figure 5:**
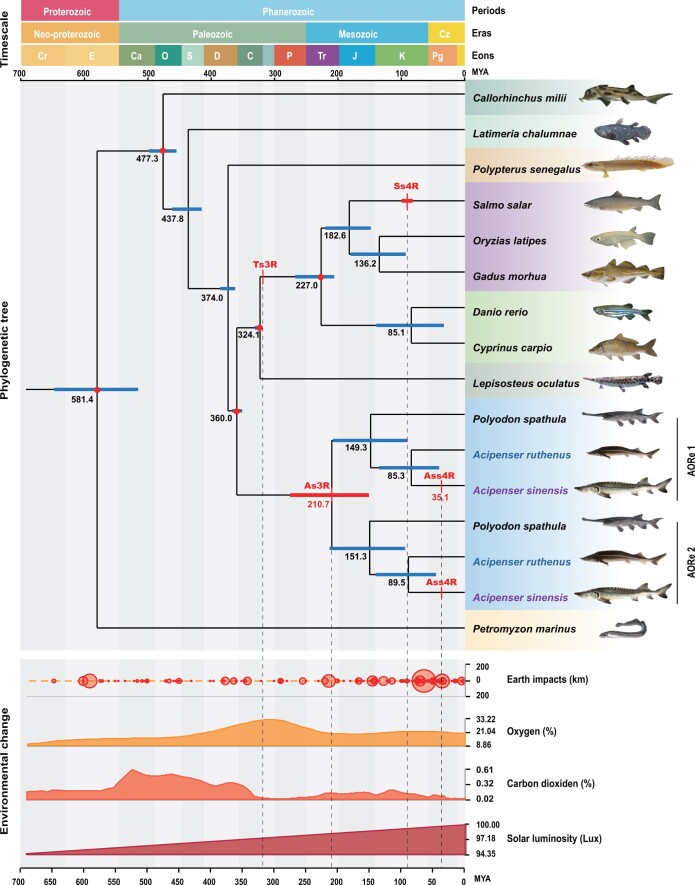
Relationship among phylogenetic tree, WGD events, and environmental changes Five red nodes are the intrinsic parameters based on fossil records. The black number indicates the divergence time. The blue blocks are the interval of estimated time. The red blocks indicate WGD time. Environmental changes display Earth impacts, oxygen, carbon dioxide, and solar luminosity in history. Cz, Cenozoic; Cr, Cryogenian; E, Ediacaran; Ca, Cambrian; O, Ordovician; S, Silurian; D, Devonian; C, Carboniferous; P, Permian; Tr, Triassic; J, Jurassic; K, Cretaceous; Pg, Paleogene; Ts3R, teleost-specific third-round WGD; As3R, Acipenseriformes-specific third-round WGD; Ss4R, salmonids-specific fourth-round WGD; Ass4R, *Acipenser sinensis*-specific fourth-round WGD; MYA, million years ago.

LORe cannot accurately reflect WGD due to delayed differentiation of LORe; however, AORe can better reflect the evolutionary trajectory of Acipenseriformes. The phylogenetic tree using MCMCTree analysis based on protein sequences of AORe in Acipenseriformes and other species ohnologues showed that the As3R occurred 210.7 MYA. Divergence of paddlefish and sturgeon occurred ∼ 150 MYA. *A*. *sinensis* and *A*. *ruthenus* diverged 89.5–85.3 MYA (**Figure 5**), which is slightly later than the 121.3 MYA (76.7–166.2 MYA) estimated by a previous report based on a mitochondrial genome sequence dataset of Acipenseriformes [33].

## Discussion

The absence of the whole-genome information of *A*. *sinensis* has hindered its genetics and evolution investigations, both of which could aid in conservation efforts and provide more information on fish ploidy evolution. *De novo* genome assembly of *A*. *sinensis* has been challenging because of the large size, higher polyploidy level, higher chromosome number, and complex chromosomal composition. In this study, we successfully obtained a high-quality chromosome-level assembly of the *A*. *sinensis* genome. This is the first sequenced octoploid sturgeon genome in the order of Acipenseriformes and also the first sequenced genome of an octoploid vertebrate to date.

From our work presented here, the assembled genome size of *A*. *sinensis* is equal to the haploid genome size of *A*. *ruthenus* [11] and *P*. *spathula* [26]. The Hi-C anchoring rate of *A*. *sinensis* (98.3%) in this study is higher than those of *A*. *ruthenus* (90.2%) and *P*. *spathula* (96.5%) (Table 1). Moreover, high coverage of the complete genome indicates high integrity of the genome assembly. Genome completeness assessment shows that the proportion of complete BUSCOs (95.6%) is higher than those in the genomes of *P*. *spathula* (93.7%) [26], *S*. *salar* (90.12%) [44], and *C*. *carpio* (81.70%) [37,38], as well as that in the first *A*. *ruthenus* genome (81.6%) [30], but slightly lower than that in the second *A*. *ruthenus* genome (98.3%) [11]. In addition, all chromosomes, especially the six macro-chromosomes, have high collinearity with *A*. *ruthenus* and *P*. *spathula*. Compared with recently reported high-quality genome assemblies of polyploid fishes, our *A*. *sinensis* genome assembly (contig N50: 4.07 Mb) has higher assembly quality as measured by contig N50 in comparison to the published tetraploid genomes of *A*. *ruthenus* (contig N50: 597.52 kb) [11] and *P*. *spathula* (contig N50: 3.44 Mb) [26], and is close to that of hexaploid Prussian carp *Carassius gibelio* (contig N50: 4.3 Mb) [40]. According to the common diploid genome assembly, 132 chromosomes are theoretically supposed to be assembled for *A*. *sinensi*s. However, the homologous copies of each chromosome result in the collapse of polyploid genome assemblies, thus forming a “mosaic” reference from both parental haplotypes as a “monoploid” representation of the genome. For the autooctoploid *A*. *sinensis*, we are able to construct 66 chromosomes based on the final assembly. This includes 6 macro-chromosomes and 60 medium/micro-chromosomes, which correspond to 1/4 of the total number of chromosomes or two monoploids of this species. Overall, while we will of course focus future work on reconstructing the remaining chromosomes, which presents significant challenges due to the complexity of the genome, we consider our current genome assembly of *A*. *sinensis* to be of high quality for an octoploid species with such a high DNA content and complex chromosome compositions.

WGS provides a powerful solution for revealing ploidy composition and evolution. Smudgeplot analyses on the basis of our WGS data show that *A*. *sinensis* and *A*. *ruthenus* ploidies are not well-defined octoploids or tetraploids as previously thought. Rather, they exhibit a complex transition ploidy with multiple compositions arising from octoploid rediploidization. We observe that *A*. *ruthenus* has five different ploidy compositions and propose that the species is a tetraploid with a certain degree of rediploidization, which is consistent with a previous study of segmental rediploidization [11]. In contrast, we find that *A*. *sinensis* has eight more complex ploidy compositions than *A*. *ruthenus* and should therefore be considered a paleooctoploid experiencing diploidization. These results are consistent with our SNP analyses in this study and previous reports using cytogenetic and molecular methods [18,22–24]. These conclusions potentially support the latest viewpoint that ploidy Groups A and B are evolutionary tetraploid and octoploid, respectively [17–19].

We further explored whether *A*. *sinensis* and *A*. *ruthenus* underwent homologous or heterologous polyploidization, respectively. Based on the Smudgeplot analyses of four representative tetraploid species, we propose a criterion for distinguishing between homologous and heterologous WGDs. Our results show that *A*. *sinensis* and *A*. *ruthenus* have striking autopolyploid characteristics based on this criterion. Burst of distinctive TEs might be closely related to separating from two subgenomes in the allopolyploid genomes. We thus also carried out a comparison of the TE landscape of *A*. *sinensis* paralogous chromosomes to explore whether *A*. *sinensis* is homologous or heterologous. For allopolyploidy, the fast-evolving repeats and relics of mobile elements are specific to their allopolyploid ancestors and thus have significant differences, whereas for an autopolyploid chromosome set, the repeat elements would not differentiate the homologs and thus the individual TE families in paralogous chromosomes were monophyletic [11,26]. We did not detect significantly specific TE families in the *A*. *sinensis* genome in this study. Our results are similar to the analysis of the *A*. *ruthenus* genome [11] and therefore, we conclude that *A*. *sinensis* is likely to be an autooctoploid.

Phylogenetic trees have shown that the Acipenseriformes ancestor diverged from the teleost ancestor ∼ 360 MYA, which is earlier than the teleost-specific WGD (Ts3R) occurring ∼ 320–350 MYA, thereby implying that Acipenseriformes experienced an independent WGD [45]. In previous reports [11,26], WGD and divergence time of Acipenseriformes were calculated based on single-copy genes or homologous genes by WGS. For example, Cheng et al. [26] reported that paddlefish (Polyodontidae) and sturgeon (Acipenseridae) diverged ∼ 81.5 MYA and a round of WGD event in the American paddlefish occurred ∼ 46.6–54.1 MYA, implying that this WGD event independently occurred in the American paddlefish after the species diverged. In contrast, Du et al. [11] reported that the *A*. *ruthenus* WGD occurred ∼ 180 MYA. We feel that it is more reasonable to calculate WGD and divergence time only based on AORe to eliminate the LORe interference. By integrating AORe, our results show that Acipenseriformes share a common WGD event (As3R) dating back to 210.7 MYA before the divergence of Acipenseridae and Polyodontidae ∼ 150 MYA (149.3–151.3 MYA), and *A*. *sinensis* underwent an additional lineage-specific WGD (Ass4R) ∼ 35.12 MYA, which resulted in the speciation of an autooctoploid species. Interestingly, a similar study on WGD based on LORe and AORe using the *A*. *ruthenus* and *P*. *spathula* genomes was performed by Redmond and his colleagues [46]. In their work, they screened a total of 5439 gene families containing high-confidence ohnolog pairs in two species, *A*. *ruthenus* and *P*. *spathula*, to analyze maximum likelihood (ML) gene trees. In comparison, we screened 1438 gene families from 4 species including *A*. *sinensis*, *A*. *ruthenus*, *P*. *spathula*, and *L*. *oculatus*. Their study excluded independent WGDs and found a high proportion of tetraploidy (∼ 50%–66% of the genome) at the time of speciation, which is inconsistent with past studies inferring independent WGD events [26,30,47–49]. Consistent with our estimates of WGD timing, Redmond et al. inferred a divergence time of ∼ 171.6 MYA (95% credibility interval range: ∼ 124.1–203.3 MYA) for the split of sturgeons and paddlefishes, and estimated a lower bound for the shared sturgeon–paddlefish WGD (As3R) at ∼ 254.7 MYA (95% credibility interval range: ∼ 207.1–289 MYA) [46]. Excitingly, the As3R time is close to the report (∼ 180 MYA) by Du et al. [11], and the divergence time is consistent with the estimated time (141.4 MYA) based on mitochondrial genome sequence datasets by Peng and his colleagues [33]. Thus, we believe that the WGD and divergence time reported here are particularly robust. Analyses based on AORe of excluded LORe interference, therefore, provide a novel method for calculating more reasonable WGD and divergence time for polyploid species.

WGD events are strongly correlated with the timing of drastic environmental changes [50], such as Earth impacts and dramatic changes in oxygen concentration, carbon dioxide concentration, or temperature [51]. Interestingly, the larger events of Earth impacts in geological history correspond well to As3R and Ass4R in this study (Figure 5), suggesting that the violently climatic and geological changes caused by Earth impacts potentially resulted in two rounds of WGDs by affecting the reproductive process of *A. sinensis* ancestors.

Overall, in this study, we accomplished the WGS of the first octoploid fish and revealed the specific ploidy composition and WGD evolutionary history of *A. sinensis*. This high-quality genome resource will serve as a powerful platform for the studies of genetics, evolution, and conservation in Acipenseriformes species, as well as provide a reference for the genomic studies of other polyploid vertebrates.

## Materials and methods

A complete description of materials and methods can be found in File S1.

### *A*. *sinensis* animals used in this study

All *A*. *sinensis* animals used in this study were derived from an artificially bred stock. A male gynogenetic *A*. *sinensis* was used for genome sequencing and assembly. An animal that was derived through sexual reproduction and normal development rather than meiotic gynogenesis was sampled for ploidy estimation and transcriptome sequencing.

### Genome and transcriptome sequencing

The genomic DNA for sequencing was extracted from the blood of a gynogenetic progeny of *A*. *sinensis* (male, 3 years old) (Figure S13). The short reads were sequenced using three paired-end (PE) libraries (170 bp, 500 bp, and 800 bp) using the Illumina HiSeq 2000 platform. We applied rigorous criteria to filter the raw reads generated by PE libraries into clean reads using SOAPfilter in the SOAPdenovo package [52,53]. The single-molecule long reads were sequenced using 8 libraries using the PacBio Sequel sequencing platform. A total of 42 single-molecule real-time (SMRT) cells were sequenced using the 20-kb large insert size libraries for genome assembly. The large genomic DNA from the fresh blood sample of *A*. *sinensis* was used for the Hi-C library construction. The extracted DNA in length of 300–350 bp was sequenced on a BGISEQ-500 sequencing platform.

A mixed sample containing 11 different tissues was sequenced using the PacBio Sequel sequencing platform. In addition, nine samples of three different tissues, containing the hypothalamus, pituitary, and gonad, from three female control individuals (normally sexual reproduction) were used for RNA-seq by the Illumina platform [54].

### Genome size estimation

The genome size of *A*. *sinensis* was estimated by flow cytometry of red blood cells from a normal reproductive animal. Three different *k*-mer analyses (19-, 21-, and 23-mer) were also implemented using Jellyfish [55] by genomic clean reads within small insert size libraries to predict the genome size. The total genome size was estimated according to the following formula: genome size = *k*-mer number/peak depth, where *k*-mer number is the total number of *k*-mers, and peak depth is the maximal frequency.

### Genome assembly and chromosome anchoring

PacBio sequencing raw data were corrected using Canu (https://github.com/marbl/canu) software. The corrected PacBio reads were assembled into original contigs using SMARTdenovo (https://github.com/ruanjue/smartdenovo). The original contigs were corrected and polished using Arrow [56] and Pilon [57] with PacBio data and Illumina HiSeq data, respectively. Furthermore, Purge_Dups (https://github.com/dfguan/purge_dups) was implemented to break misjoins and generate a final assembly. The contigs were then anchored to chromosomes using Juicer (https://github.com/aidenlab/juicer) and 3D-DNA (https://github.com/aidenlab/3d-dna) pipelines with the Hi-C data. SMARTdenovo has demonstrated notable effectiveness in handling polyploid and highly heterozygous genome assemblies. Arrow and Pilon are capable of effectively correcting erroneous nucleotide bases. Purge_Dups is also a commonly employed method for filtering redundant sequences and obtaining haploid assemblies.

### Genome annotation

We predicted genes in the genome of *A*. *sinensis* using *ab initio*-based, homology-based, and transcriptome-assisted methods. *De novo* gene prediction was performed using AUGUSTUS and SNAP. The protein sequences of *C*. *milii*, *D*. *rerio*, *L*. *chalumnae*, *L*. *oculatus*, and *P*. *marinus* (Ensembl release 100 version) were downloaded from the Ensembl database for homology-based gene set prediction by Exonerate software. Gene structures were annotated using three approaches (*ab initio* predictions, homologous proteins, and transcriptome data) that were combined using MAKER software. Furthermore, gene functions were annotated against seven public databases including the NR, Swiss-Prot, KEGG, KOG, TrEMBL, InterPro, and GO databases according to the best match of the alignments using BLASTp (E-value < 1 × 10^−5^).

Two kinds of repeats, tandem repeats and TEs, were identified before performing genome annotation. Tandem repeats were predicted using Tandem Repeats Finder (v4.09) [58]. TEs were detected based on homology and *de novo* strategies. For the homology approach, TEs were predicted using RepeatMasker [59] and RepeatProteinMask based on the Repbase database [60] and the TE database in the RepeatMasker software package, respectively. For the *de novo* approach, the *de novo* repeat library was predicted using RepeatModeler (RepeatModeler-open-1.0.11; http://www.repeatmasker.org/RepeatModeler/), and TEs were annotated by RepeatMasker software based on the *de novo* library.

### Collinearity analysis, gene family identification, and phylogenetic tree construction

MCscan (Python version) [61] was used for the genomic analysis between *L. oculatus*, *P.*  *spatula*, *A. ruthenus*, and *A*. *sinensis*. The collinearity figure was drawn based on the collinear gene pair information between species.

Thirteen species (*A*. *sinensis*, *A*. *ruthenus*, *C*. *milii*, *D*. *rerio*, *G*. *morhua*, *L*. *chalumnae*, *L*. *oculatus*, *O*. *latipes*, *P*. *marinus*, *P*. *spathula*, *P*. *senegalus*, *C*. *carpio*, and *S*. *salar*) were used in the phylogenetic analysis. The protein-coding genes of these species were downloaded and filtered, and only the longest open reading frame (ORF) with a gene encoding more than 50 amino acids was remained for the gene family clustering and phylogenetic analysis. Because of the rediploidization of *P*. *spathula*, *A*. *sinensis*, and *A*. *ruthenus*, the protein-coding genes were separated into two haplotype sets in the following analysis process. Gene families were identified with OrthoFinder [62]. The single-copy orthologous genes from gene families were further aligned using MUSCLE (RRID: SCR_011812; v3.8.31) [63] with default parameters and subsequently translated reversely into codon sequences. These aligned sequences were concatenated to generate a super alignment matrix for phylogenetic reconstruction based on PhyML (RRID: SCR_014629) [31] with four-fold degenerate (4D) sites of the single-copy orthologs shared among the 13 species. Additionally, we employed IQ-TREE (v1.6.12) [64] to construct gene phylogenetic trees of the single-copy orthologs and used ASTRAL (v5.6.1; https://github.com/maryamrabiee/Constrained-search) to integrate the gene trees. The resulting phylogenetic tree was consistent with the tree generated by PhyML. The divergence time was determined by MCMCTree implemented in the Phylogenetic Analysis by Maximum Likelihood (PAML; RRID: SCR_014932; v4.5) package [65,66] with the approximate likelihood calculation method, the correlated molecular clock, and the general reversible (REV) substitution model, successively. Three divergence dates from the TimeTree database [67] were used for calibration.

### Ploidy evaluation

Ploidy of the species was estimated by the maximum number of alleles per individual at each microsatellite locus. To obtain available SSR markers for determining the ploidy of *A*. *sinensis*, all screened SSRs of a tetra-nucleotide repeat were verified by polyacrylamide gel electrophoresis (PAGE) and capillary electrophoresis on the Applied Biosystems Genetic Analyzer (Catalog No. ABI 3730, Thermo Fisher Scientific, Foster City, CA), respectively.

PE reads of *A*. *sinensis* and *A*. *ruthenus* were mapped to their assembled scaffolds by aligner BWA (v0.7.12-r1039) and SAMtools (v1.4). The heterozygous SNPs were called by FreeBayes (v0.9.10-3-g47a713e). The average allele mapping depth and the minor allele frequency of the variant sites were calculated to estimate ploidy based on the heterozygous sequence polymorphism.

### Heterozygous *k*-mer pair analysis

To disentangle the genomic ploidy of *C*. *carpio*, *T*. *arcticus*, *M*. *sativa*, *A*. *sinensis*, and *A*. *ruthenus*, we extracted the haplotype structures from heterozygous *k*-mer pairs by using the Smudgeplot pipeline [68]. First, we produced a *k*-mer frequency file by KMC [69] with *k* = 21 from trimmed reads. Then, we searched for all heterozygous *k*-mer pairs that differed at exactly one nucleotide through a systematic scan of all input *k*-mers. To avoid sequencing errors with genomic *k*-mers, we filtered the *k*-mers with a depth of less than 14, which was the depth of the first trough in the *k*-mer frequency curve. Finally, we performed the R script of the pipeline, plotted the Smudgeplot, and estimated ploidy using the coverage file of heterozygous *k*-mers. This tool performed gymnastics with the heterozygous *k*-mer pairs by comparing the sum of *k*-mer pair coverages (CovA + CovB) to their relative coverage [CovA/(CovA + CovB)].

### LORe and AORe analyses

Combined with gene family identification and genome collinearity analysis, we identified the potential ohnologues with 2:2:2:1 in *A*. *sinensis*, *A*. *ruthenus*, *P*. *spathula*, and *L*. *oculatus.* The protein sequences of ohnologues were aligned using MUSCLE (v3.8.425) [63] with the default parameters. These alignments were subsequently converted into coding sequence (CDS) alignment by tracing the coding relationship using PAL2NAL (v14) [70]. Gblocks (v0.91b) [71] was employed to conduct further checks (trim) on the CDS alignments with parameters “-t = c”. The trimmed alignments with length less than 150 bp were filtered and then transmitted to IQ-TREE (v1.6.12) [64] to infer the gene tree with settings: -alrt 1000 -bb 1000. Each trimmed gene was subjected to a gene tree analysis in the same manner. DensiTree [72] was used to visualize the topologies of these trees.

The protein sequences of AORe ohnologues were selected. The ohnologues of AORe exhibited a pairable topology of PSR-PSR, as inferred from the aforementioned analysis of three species. The protein sequences of AORe ohnologues were aligned using MUSCLE (v3.8) [63] with default parameters, and subsequently translated reversely into codon sequences. These aligned sequences were concatenated to generate a super alignment matrix, and phylogenetic reconstruction was performed using the ML method in IQ-TREE (v1.6.12) [64]. The best-fit evolutionary substitution model was determined using ModelFinder. Based on the phylogenetic tree, the divergence time between individual species and ohnologue subgenomes was estimated based on the proteins using MCMCTree implemented in the PAML (RRID: SCR_014932; v4.5) package [65,66] with the approximate likelihood calculation method, the correlated molecular clock, and the general REV substitution model, successively. Four datasets from the TimeTree database [67] were used for calibration. We calculated *Ks* of AORe ohnologues of inter-species and intra-species in the three genomes. *Ks* analysis was performed using the wgd package with default parameters and the “FastTree” node weighting method [31,63,65,73]. Log normal distributions in *Ks* were plotted based on node-averaged values as calculated in the wgd package. The Gaussian mixture models (GMMs) were fitted to the *Ks* distribution following the wgd pipeline, with the optimal number of components assessed using the Bayesian information criterion.

## Ethical statement

The experiments were performed according to the guidelines of the Institutional Review Board on Bioethics and Biosafety of the Chinese Sturgeon Research Institute (Approval No. ZHX-20131211).

## Supplementary Material

qzad002_Supplementary_Data

## Data Availability

The assembled genome sequences and gene annotations have been deposited in the Genome Warehouse [74] at the National Genomics Data Center (NGDC), Beijing Institute of Genomics (BIG), Chinese Academy of Sciences (CAS) / China National Center for Bioinformation (CNCB) (GWH: GWHBQEF00000000), and are publicly accessible at https://ngdc.cncb.ac.cn/gwh. The raw datasets for genome assembly have been deposited in the Genome Sequence Archive [75] at the NGDC, BIG, CAS / CNCB (GSA: CRA009603), and are publicly accessible at https://ngdc.cncb.ac.cn/gsa.

## CRediT author statement

**Binzhong Wang:** Conceptualization, Investigation, Formal analysis, Data curation, Visualization, Writing – original draft, Writing – review & editing. **Bin Wu:** Conceptualization, Formal analysis, Investigation, Data curation, Writing – original draft, Writing – review & editing. **Xueqing Liu:** Investigation, Resources. **Yacheng Hu:** Resources, Validation. **Yao Ming:** Writing – review & editing. **Mingzhou Bai:** Investigation. **Juanjuan Liu:** Investigation, Resources, Writing – review & editing. **Kan Xiao:** Resources. **Qingkai Zeng:** Conceptualization, Resources. **Jing Yang:** Writing – review & editing. **Hongqi Wang:** Methodology. **Baifu Guo:** Resources. **Chun Tan:** Resources. **Zixuan Hu**: Writing – review & editing. **Xun Zhao:** Resources. **Yanhong Li:** Investigation, Methodology. **Zhen Yue**: Methodology. **Junpu Mei**: Methodology. **Wei Jiang:** Supervision. **Yuanjin Yang**: Resources. **Zhiyuan Li**: Supervision. **Yong Gao:** Conceptualization, Supervision. **Lei Chen:** Conceptualization, Supervision, Project administration, Funding acquisition. **Jianbo Jian:** Investigation, Data curation, Writing – review & editing. **Hejun Du:** Conceptualization, Investigation, Supervision, Writing – review & editing, Project administration, Funding acquisition. All authors have read and approved the final manuscript.

## ORCID

0000-0003-1165-2701 (Binzhong Wang)

0000-0001-7507-9970 (Bin Wu)

0000-0002-0329-1398 (Xueqing Liu)

0000-0003-4408-9150 (Yacheng Hu)

0009-0002-2379-3886 (Yao Ming)

0000-0001-5666-8200 (Mingzhou Bai)

0000-0001-9638-7075 (Juanjuan Liu)

0009-0003-6852-4548 (Kan Xiao)

0000-0001-9350-7334 (Qingkai Zeng)

0000-0003-3424-4575 (Jing Yang)

0000-0002-6986-198X (Hongqi Wang)

0009-0009-4957-1270 (Baifu Guo)

0000-0002-9483-8538 (Chun Tan)

0009-0005-4571-4730 (Zixuan Hu)

0009-0009-7394-0072 (Xun Zhao)

0000-0002-4275-0112 (Yanhong Li)

0000-0001-8760-7574 (Zhen Yue)

0000-0003-4630-0765 (Junpu Mei)

0000-0003-0287-9269 (Wei Jiang)

0009-0006-2553-8490 (Yuanjin Yang)

0009-0006-6328-0734 (Zhiyuan Li)

0000-0003-3901-1930 (Yong Gao)

0009-0000-1408-4741 (Lei Chen)

0000-0003-2187-5490 (Jianbo Jian)

0000-0002-2959-7870 (Hejun Du)
