# Supplementary material for: Whole-genome Sequencing Reveals Autooctoploidy in Chinese Sturgeon and Its Evolutionary Trajectories
Source: Genomics Proteomics Bioinformatics. 2023 Dec 13;22(1):qzad002. doi: 10.1093/gpbjnl/qzad002 (PMC11425059; doi:10.1093/gpbjnl/qzad002)
Supplement: qzad002_Supplementary_Data [file qzad002_supplementary_data.zip › Supplementary material captions.docx]

## Supplementary material

**File S1 Supplementary information**

**Figure S1 Relationships among WGD, divergence, and rediploidization based on AORe and LORe model**

**A.** The AORe model of post-WGD, and the LORe model of post-WGD evolution following delayed rediploidization. **B.** PSR-PSR, PP-SR-SR, and PP-SS-RR model. PSR-PSR was constructed by AORe and the divergence and speciation occurred after As3R and the complete rediploidization. PP-SR-SR was constructed by LORe and the two families of Acipenseriformes diverged after As3R before the complete rediploidization. PP-SS-RR was constructed by LORe and the divergence and speciation occurred before the complete rediploidization after As3R. WGD, whole-genome duplication; LORe, lineage-specific ohnologue resolution; AORe, ancestral ohnologue resoluti*o*n; As3R, the first Acipenseriforme-specific WGD / the Acipenseriforme-specific 3rd vertebrate WGD; P / Psp, *Polyodon spathula*; S / Asi, *Acipenser sinensis*; R / Aru, *Acipenser ruthenus*; Sp1, species 1; Sp2, species 2.

**Figure S2 19-mer, 21-mer, and 23-mer distributions were used for the estimation of genome size**

The X-axis represents the depth of sequencing. The Y-axis is the proportion of 17-mer frequencies at different depths. The total genome size was estimated according to the following formula: genome size = *k*-mer number / peak depth, where *k*-mer number is the total number of *k*-mers, and peak depth is the maximal frequency.

**Figure S3** [**Flow cytometer**](javascript:;) **result of *Acipenser sinensis***

**Figure S4 Venn diagram of gene function annotation based on NR, InterPro,** **KEGG, Swiss-Prot, and KOG databases**

A total of 22,939 genes were shared by the eight database annotations. NR, Non-Redundant Protein Sequence Database in National Center of Biotechnology Information; KEGG, Kyoto Encyclopedia of Genes and Genomes; KOG, EuKaryotic Orthologous Groups.

**Figure S5 Phylogenetic tree constructed with 2096 single copy orthologous genes using ASTRAL**

‘100’ is bootstrap value.

**Figure S6 Phylogenetic tree constructed with 2096 single copy orthologous genes using PhyML**

The branch length represents the neutral divergence rate. ‘100’ is bootstrap value.

**Figure S7 Karyotype of *Acipenser sinensis***

A total of 264 chromosomes were identified (left) and ranged in groups containing four chromosomes (right). The first row is the macro-chromosomes.

**Figure S8 Ploidy identification based on SSRs**

Eight peaks were identified by tetra-nucleotide repeat SSRs using capillary electrophoresis on ABI PRISM 3730 Genetic Analyzer. SSRs, simple sequence repeats.

**Figure** **S9 Detection of homology and heterology based on differential TEs screening**

Pair_blocks_1–10 at the X-axis are 10 homoeologous sequence blocks with collinearity. The Y-axis represents 20 TEs in the 10 homoeologous blocks. If at least one Nsd block appears in a row, the row is not a differential TE. TE, transposable element; LINE, long interspersed nuclear element; LTR, long terminal repeat; Nsd, the block pair of TE has no significant difference.

**Figure S10 Homologous gene dot plot within the *Acipenser sinensis* genome**

*Ks* value for homologous genes in each inferred collinear block is shown. The high *Ks* values were presented on macrochromosomes 1–6. Chr, Chromosome; *Ks*, synonymous substitution rate.

**Figure S11 Distribution of *Ks* analysis in coding genes and unitary pseudogenes**

*Ka*, non-synonymous substitution rate.

**Figure S12 Amino acid sequence of the *COG7* coding gene and pseudogene alignment in *Acipenser sinensis* and *Acipenser ruthenus***

Four genes were selected. As-Pseudogene1 (Gene ID: ACSI009096-D2), Ar-Gene1 (Gene ID: ACSI009096), As-Gene2 (Gene ID: XP_033886174.2) and Ar-Gene2 (Gene ID: XP_033907760.1) were from the common AORe, respectively. -, missing codons; *, frameshifts pointed by the red arrow; X, premature stops marked by the blue arrow.

**Figure S13 Identification results for gynogenetic *Acipenser sinensis* by microsatellite DNA analysis on** **PAGE**

M, the DNA ladder marker; 1–21, gynogenetic individuals; D, the dam (maternal); S, sire (paternal); C1–C5, the control diploid individuals; PAGE, polyacrylamide gel electrophoresis.

**Table S1 Statistics of *Acipenser sinensis* sequence data derived from paired-end and mate-paired sequencing by Illumina platform**

**Table S2 Raw data statistics of PacBio sequencing**

**Table S3 Statistics of Hi-C sequencing and genome alignment**

**Table S4 Statistics of genome size estimation by different *k*-mer analysis**

**Table S5 Statistics of chromosome relative length of *Acipener sinensis***

**Table S6 Statistics of BUSCOs estimation of *Acipenser sinensis* genome assembly**

**Table S7 Statistics of RNA-seq data**

**Table S8 BUSCOs evaluation of gene annotation**

**Table S9 Statistics of gene function annotation in different databases**

**Table S10 General statistics of repeats in the genome**

**Table S11 Statistics of TEs content in *Acipenser sinensis* genome**

**Table S12** **Syntenic gene statistics in different species groups**

**Table S13 Statistics of gene family analysis based on OrthoFinder**

**Table S14 Distribution of tetra-nucleotide repeats in the screening process**

**Table S15 Comparison of SNPs and heterozygosity**

**Table S16 Homoeologous block with collinearity for specific repeats cluster**

**Table S17 Statistics of TE families in the 10 block-pairs (bp / 1 Mb)**

**Table S18 Statistics of three topological structures based on LORe and AORe**

**Table S19 Statistics of three topological structures in large chromosomes and small chromosomes of three Acipenseriforme species**
