# Supplementary material for: Whole-genome Sequencing Reveals Autooctoploidy in Chinese Sturgeon and Its Evolutionary Trajectories
Source: Genomics Proteomics Bioinformatics. 2023 Dec 13;22(1):qzad002. doi: 10.1093/gpbjnl/qzad002 (PMC11425059; doi:10.1093/gpbjnl/qzad002)
Supplement: qzad002_Supplementary_Data [file qzad002_supplementary_data.zip › Table S1-new.docx]

| **Insert size (bp)** | **Library (Lane)** | **Platform** | **Read length (bp)** | **Raw data** | | **Clean data** | |
| --- | --- | --- | --- | --- | --- | --- | --- |
|  |  |  |  | **Total bases (Mb)** | **Sequencing depth (×)** | **Total bases (Mb)** | **Sequencing depth (×)** |
| 170 | 3 (5) | HiSeq 2000 | 100 | 212,146.46 | 54.397 | 183,776.71 | 47.122 |
| 500 | 2 (4) | HiSeq 2000 | 100 | 154,815.22 | 39.696 | 126,032.85 | 32.316 |
| 800 | 2 (4) | HiSeq 2000 | 100 | 144,048.39 | 36.935 | 111,772.17 | 28.66 |
| Total | 7 (15) |  |  | 511,010.07 | 131.03 | 421,581.73 | 108.10 |

**Table S1 Statistics of *Acipenser sinensis* sequence data derived from** **paired-end and mate-paired sequencing by Illumina platform**
