# Supplementary material for: Whole-genome Sequencing Reveals Autooctoploidy in Chinese Sturgeon and Its Evolutionary Trajectories
Source: Genomics Proteomics Bioinformatics. 2023 Dec 13;22(1):qzad002. doi: 10.1093/gpbjnl/qzad002 (PMC11425059; doi:10.1093/gpbjnl/qzad002)
Supplement: qzad002_Supplementary_Data [file qzad002_supplementary_data.zip › Table S2-by JieLiu-wbz.docx]

**Table S2 Raw data statistics of PacBio sequencing**

| **ID** | **Total bases (Gb)** | **Total reads (bp)** | **Average length (bp)** | **Max length (bp)** | **Min length (bp)** | **N50 length (bp)** |
| --- | --- | --- | --- | --- | --- | --- |
| r54040_20180502_034917-1_C01 | 4.77 | 422,713 | 11,277.01 | 76,135 | 50 | 16,766 |
| r54040_20180503_063650-1_D01 | 2.14 | 188,453 | 11,381.96 | 76,800 | 50 | 16,841 |
| r54040_20180513_033318-1_E01 | 6.3 | 1,033,967 | 6092.22 | 80,944 | 50 | 10,522 |
| r54040_20180515_095654-1_F01 | 6.64 | 1,042,772 | 6369.35 | 77,535 | 50 | 11,020 |
| r54040_20180516_093202-1_G01 | 6.63 | 867,880 | 7642 | 83,551 | 50 | 12,402 |
| r54040_20180519_035010-1_A01 | 7.85 | 832,746 | 9427.11 | 86,299 | 50 | 14,101 |
| r54040_20180521_100410-1_G01 | 5.66 | 671,653 | 8425.29 | 76,645 | 50 | 13,024 |
| r54040_20180521_100410-2_H01 | 5.87 | 705,141 | 8327.71 | 81,130 | 50 | 12,876 |
| r54040_20180523_082053-1_A01 | 6.95 | 734,602 | 9466.02 | 81,221 | 50 | 14,842 |
| r54040_20180523_082053-2_B01 | 6.34 | 629,034 | 10,083.05 | 81,901 | 50 | 15,298 |
| r54160_20180511_073108-1_F01 | 4.94 | 503,854 | 9803.66 | 74,420 | 50 | 14,352 |
| r54160_20180511_073108-2_G01 | 4.98 | 534,022 | 9323.51 | 70,144 | 50 | 13,832 |
| r54160_20180517_100324-1_H01 | 6.33 | 791,426 | 8001.82 | 80,397 | 50 | 12,855 |
| r54160_20180521_100436-1_A01 | 6.99 | 825,058 | 8471.55 | 85,626 | 50 | 13,069 |
| r54160_20180521_100436-2_B01 | 5.85 | 725,603 | 8057.1 | 78,546 | 50 | 12,616 |
| r54160_20180523_081910-1_C01 | 5.38 | 526,650 | 10,214.24 | 86,225 | 50 | 15,344 |
| r54160_20180523_081910-2_D01 | 7.13 | 768,591 | 9275.9 | 93,127 | 50 | 14,639 |
| r54254_20180518_100604-1_A01 | 6.36 | 774,771 | 8203.37 | 77,108 | 50 | 12,946 |
| r54254_20180518_100604-2_B01 | 5.73 | 729,806 | 7845.24 | 86,221 | 50 | 12,557 |
| r54263_20180504_045317-1_E01 | 5.85 | 520,621 | 11,230.49 | 73,109 | 50 | 17,015 |
| r54263_20180504_045317-2_F01 | 1.87 | 170,469 | 10,981.08 | 74,645 | 50 | 16,265 |
| r54263_20180512_083616-1_H01 | 5.87 | 934,289 | 6285.93 | 78,853 | 50 | 10,933 |
| r54263_20180522_113709-1_H01 | 7.33 | 746,669 | 9818.5 | 87,009 | 50 | 15,011 |
| r54266_20180505_092109-1_A01 | 3.35 | 318,649 | 10,514.71 | 85,314 | 50 | 14,708 |
| r54266_20180511_073539-1_D01 | 1.99 | 171,355 | 11,596.01 | 79,424 | 50 | 17,454 |
| r54267_20180430_081513-1_A01 | 3.24 | 271,369 | 11,921.08 | 76,085 | 50 | 17,352 |
| r54267_20180505_092334-1_A01 | 4.09 | 338,988 | 12,052.17 | 78,067 | 50 | 17,498 |
| r54267_20180506_042647-1_B01 | 3.53 | 314,237 | 11,238.17 | 76,498 | 50 | 16,634 |
| r54267_20180508_080551-1_G01 | 5.8 | 636,383 | 9106.98 | 74,451 | 50 | 14,183 |
| r54267_20180520_065003-1_E01 | 6.74 | 801,576 | 8413.85 | 82,834 | 50 | 13,126 |
| r54267_20180520_065003-2_F01 | 6.59 | 798,957 | 8243.27 | 82,663 | 50 | 12,931 |
| r54268_20180520_065520-1_C01 | 6.41 | 738,444 | 8674.6 | 80,897 | 50 | 13,367 |
| r54268_20180520_065520-2_D01 | 5.95 | 694,523 | 8564.32 | 85,089 | 50 | 13,200 |
| r54269_20180506_042927-1_B01 | 6.37 | 621,436 | 10,246.23 | 85,142 | 50 | 14,427 |
| r54269_20180507_095115-1_C01 | 6.32 | 629,217 | 10,039.05 | 77,396 | 50 | 14,156 |
| r54269_20180509_083440-1_D01 | 4.82 | 928,344 | 5194.33 | 65,871 | 50 | 9516 |
| r54270_20180501_045852-1_B01 | 3.3 | 292,680 | 11,286.51 | 79,195 | 50 | 17,329 |
| r54270_20180510_070646-1_E01 | 3.03 | 290,342 | 10,443.22 | 82,116 | 50 | 15,199 |
| r54270_20180515_100253-1_G01 | 5.04 | 654,895 | 7696.18 | 71,507 | 50 | 11,182 |
| r54270_20180516_093557-1_B01 | 5.59 | 809,653 | 6905.92 | 73,590 | 50 | 10,529 |
| r54272_20180509_083649-1_H01 | 4.17 | 559,657 | 7444.11 | 72,862 | 50 | 12,364 |
| r54272_20180510_070828-1_C01 | 1.87 | 169,603 | 11,000.46 | 76,899 | 50 | 16,546 |
