# Supplementary material for: Whole-genome Sequencing Reveals Autooctoploidy in Chinese Sturgeon and Its Evolutionary Trajectories
Source: Genomics Proteomics Bioinformatics. 2023 Dec 13;22(1):qzad002. doi: 10.1093/gpbjnl/qzad002 (PMC11425059; doi:10.1093/gpbjnl/qzad002)
Supplement: qzad002_Supplementary_Data [file qzad002_supplementary_data.zip › Table S3-by JieLiu-wbz.docx]

**Table S3 Statistics of Hi-C sequencing and genome alignment**

|  | **Paired-end reads number** | **Data size (Gb)** | **Proportion (%)** |
| --- | --- | --- | --- |
| Sequenced read pairs | 3,998,723,270 | 799.74 | 100 |
| Normal paired | 1,284,694,689 | 256.94 | 32.13 |
| Chimeric paired | 253,009,088 | 50.60 | 6.33 |
| Alignable paired | 1,537,703,777 | 307.54 | 38.45 |
| Hi-C contacts paired | 864,335,190 | 172.87 | 21.61 |
| Inter-chromosomal paired | 500,555,919 | 100.11 | 12.51 |
| Intra-chromosomal paired | 363,779,271 | 72.76 | 9.10 |

*Note*: Hi-C, high-through chromosome conformation capture.
