# Supplementary material for: Whole-genome Sequencing Reveals Autooctoploidy in Chinese Sturgeon and Its Evolutionary Trajectories
Source: Genomics Proteomics Bioinformatics. 2023 Dec 13;22(1):qzad002. doi: 10.1093/gpbjnl/qzad002 (PMC11425059; doi:10.1093/gpbjnl/qzad002)
Supplement: qzad002_Supplementary_Data [file qzad002_supplementary_data.zip › Table S4-by JieLiu by Chi by wbz.docx]

**Table S4 Statistics of genome size estimation by different *k*-mer analysis**

| **Genome** | ***k*** | ***k*-mer number** | **Peak depth** | **Genome size** | **Number of used bases** | **Number of used reads** | **Read depth (×)** |
| --- | --- | --- | --- | --- | --- | --- | --- |
| ***Acipenser sinensis*** | 21 | 341,727,680,524 | 46 | 7,428,862,620 | 408,658,516,300 | 4,086,585,163 | 55.01 |
|  | 21 | 341,727,680,524 | 92 | 3,714,431,310 | 408,658,516,300 | 4,086,585,163 | 110.02 |
|  | 21 | 341,727,680,524 | 173 | 1,975,304,512 | 408,658,516,300 | 4,086,585,163 | 206.89 |

*Note*: The genome size was estimated according to the formula: Genome size = *k*-mer number / peak depth. Read depth = number of used bases / genome size.
