# Supplementary material for: Whole-genome Sequencing Reveals Autooctoploidy in Chinese Sturgeon and Its Evolutionary Trajectories
Source: Genomics Proteomics Bioinformatics. 2023 Dec 13;22(1):qzad002. doi: 10.1093/gpbjnl/qzad002 (PMC11425059; doi:10.1093/gpbjnl/qzad002)
Supplement: qzad002_Supplementary_Data [file qzad002_supplementary_data.zip › Table S6-by JieLiu by Chi by wbz.docx]

**Table S6 Statistics of BUSCOs estimation of *Acipenser sinensis* genome assembly**

| **Type** | **Number** | **Percentage (%)** |
| --- | --- | --- |
| Complete BUSCOs (C) | 3207 | 95.62 |
| Complete and single-copy BUSCOs (S) | 2014 | 60.05 |
| Complete and duplicated BUSCOs (D) | 1193 | 35.57 |
| Fragmented BUSCOs (F) | 72 | 2.15 |
| Missing BUSCOs (M) | 75 | 2.23 |
| Total BUSCO groups searched | 3354 |  |
