# Supplementary material for: Whole-genome Sequencing Reveals Autooctoploidy in Chinese Sturgeon and Its Evolutionary Trajectories
Source: Genomics Proteomics Bioinformatics. 2023 Dec 13;22(1):qzad002. doi: 10.1093/gpbjnl/qzad002 (PMC11425059; doi:10.1093/gpbjnl/qzad002)
Supplement: qzad002_Supplementary_Data [file qzad002_supplementary_data.zip › Table S7-by JieLiu by Chi-wbz.docx]

**Table S7 Statistics of RNA-seq data**

| **Total number** | **Total length (bp)** | **N50 (bp)** | **N90 (bp)** | **Max length (bp)** | **Min length (bp)** | **Sequence GC (%)** |
| --- | --- | --- | --- | --- | --- | --- |
| 97,393 | 116,994,615 | 1530 | 612 | 7425 | 297 | 51.89 |

*Note*: RNA-seq, RNA sequencing.
