# Supplementary material for: Whole-genome Sequencing Reveals Autooctoploidy in Chinese Sturgeon and Its Evolutionary Trajectories
Source: Genomics Proteomics Bioinformatics. 2023 Dec 13;22(1):qzad002. doi: 10.1093/gpbjnl/qzad002 (PMC11425059; doi:10.1093/gpbjnl/qzad002)
Supplement: qzad002_Supplementary_Data [file qzad002_supplementary_data.zip › Table S8-by JieLiu by Chi-wbz.docx]

**Table S8 BUSCOs evaluation of gene annotation**

| **Type** | ***Acipenser sinensis*** | |
| --- | --- | --- |
|  | **Number** | **Proportion (%)** |
| Complete | 3124 | 93.1 |
| Complete and single-copy | 2184 | 65.1 |
| Complete and duplicated | 940 | 28 |
| Fragmented | 92 | 2.7 |
| Missing | 138 | 4.2 |
| Total BUSCO groups searched | 3354 | 100 |
