# Supplementary material for: Whole-genome Sequencing Reveals Autooctoploidy in Chinese Sturgeon and Its Evolutionary Trajectories
Source: Genomics Proteomics Bioinformatics. 2023 Dec 13;22(1):qzad002. doi: 10.1093/gpbjnl/qzad002 (PMC11425059; doi:10.1093/gpbjnl/qzad002)
Supplement: qzad002_Supplementary_Data [file qzad002_supplementary_data.zip › Table S9-by JieLiu by Chi-wbz.docx]

**Table S9 Statistics of gene function annotation in different databases**

| **Values** | **Total** | **NR** | **Swiss-Prot** | **KEGG** | **KOG** | **TrEMBL** | **InterPro** | **GO** | **Overall** |
| --- | --- | --- | --- | --- | --- | --- | --- | --- | --- |
| Number | 36,837 | 33,808 | 30,340 | 28,799 | 25,451 | 33,587 | 31,021 | 21,297 | 34,950 |
| Proportion (%) | 100 | 91.78 | 82.36 | 78.18 | 69.09 | 91.18 | 84.21 | 57.81 | 94.88 |

*Note*: NR, Non-Redundant Protein Sequence Database; KEGG, Kyoto Encyclopedia of Genes and Genomes; KOG, EuKaryotic Orthologous Groups; TrEMBL, Translation of European Molecular Biology Laboratory; GO, Gene Ontology.
