# Supplementary material for: Whole-genome Sequencing Reveals Autooctoploidy in Chinese Sturgeon and Its Evolutionary Trajectories
Source: Genomics Proteomics Bioinformatics. 2023 Dec 13;22(1):qzad002. doi: 10.1093/gpbjnl/qzad002 (PMC11425059; doi:10.1093/gpbjnl/qzad002)
Supplement: qzad002_Supplementary_Data [file qzad002_supplementary_data.zip › Table S10-by JieLiu-wbz.docx]

**Table S10 General statistics of repeats in the genome**

| **Type** | **Repeat size** | **Proportion in the genome (%)** |
| --- | --- | --- |
| Tandem Repeats Finder | 204,277,919 | 10.2376 |
| RepeatMasker | 281,811,105 | 14.1232 |
| ProteinMask | 196,600,118 | 9.8528 |
| *De novo* | 905,712,834 | 45.3906 |
| Total | 991,351,527 | 49.6825 |
