# Supplementary material for: Whole-genome Sequencing Reveals Autooctoploidy in Chinese Sturgeon and Its Evolutionary Trajectories
Source: Genomics Proteomics Bioinformatics. 2023 Dec 13;22(1):qzad002. doi: 10.1093/gpbjnl/qzad002 (PMC11425059; doi:10.1093/gpbjnl/qzad002)
Supplement: qzad002_Supplementary_Data [file qzad002_supplementary_data.zip › Table S11-by JieLiu by Chi-wbz.docx]

**Table S11 Stactistics of TEs content in *Acipenser sinensis* genome**

| **Type** | **Repbase TEs** | |  | **TE protiens** | |  | ***De novo*** | |  | **Combined TEs** | |
| --- | --- | --- | --- | --- | --- | --- | --- | --- | --- | --- | --- |
|  | **Length (bp)** | **Proportion in genome (%)** |  | **Length (bp)** | **Proportion in genome (%)** |  | **Length (bp)** | **Proportion in genome (%)** |  | **Length (bp)** | **Proportion in genome (%)** |
| DNA | 121,265,006 | 6.0773 |  | 29,858,845 | 1.4964 |  | 294,234,678 | 14.7458 |  | 352,537,518 | 17.6677 |
| LINE | 99,881,625 | 5.0057 |  | 100,209,084 | 5.0221 |  | 276,113,384 | 13.8377 |  | 326,045,590 | 16.3401 |
| SINE | 8,310,617 | 0.4165 |  | 0 | 0.0000 |  | 30,139,426 | 1.5105 |  | 37,569,933 | 1.8829 |
| LTR | 62,703,787 | 3.1425 |  | 66,593,825 | 3.3374 |  | 329,287,862 | 16.5026 |  | 347,742,226 | 17.4274 |
| Other | 134,796 | 0.0068 |  | 0 | 0.0000 |  | 1,704,397 | 0.0854 |  | 1,835,079 | 0.0920 |
| Unknown | 0 | 0.0000 |  | 0 | 0.0000 |  | 2,372,463 | 0.1189 |  | 2,372,463 | 0.1189 |
| Total | 281,811,105 | 14.1232 |  | 196,600,118 | 9.8528 |  | 871,279,062 | 43.6649 |  | 903,496,192 | 45.2795 |

*Note*: TE, transposable element; LINE, long interspersed nuclear element; SINE, short interspersed nuclear element; LTR, long terminal repeats.
