# Supplementary material for: Whole-genome Sequencing Reveals Autooctoploidy in Chinese Sturgeon and Its Evolutionary Trajectories
Source: Genomics Proteomics Bioinformatics. 2023 Dec 13;22(1):qzad002. doi: 10.1093/gpbjnl/qzad002 (PMC11425059; doi:10.1093/gpbjnl/qzad002)
Supplement: qzad002_Supplementary_Data [file qzad002_supplementary_data.zip › Table S12-by JieLiu by Chi by wbz by Chi.docx]

**Table S12 Syntenic gene statistics in different species groups**

| **Group** | **Species 1** | **Species 2** | **Number of sythenic genes** |
| --- | --- | --- | --- |
| As–As | *Acipenser sinensis* | *Acipenser sinensis* | 25,687 |
| As–Ar | *Acipenser sinensis* | *Acipenser ruthenus* | 31,348 |
| As–Ps | *Acipenser sinensis* | *Polyodon spathula* | 28,945 |
| As–Lo | *Acipenser sinensis* | *Lepisosteus oculatus* | 16,062 |
| Ar–Ps | *Acipenser ruthenus* | *Polyodon spathula* | 34,031 |
| Ps–Lo | *Polyodon spathula* | *Lepisosteus oculatus* | 18,053 |

*Note*: As, *Acipenser sinensis*; Ar, *Acipenser ruthenus*; Ps, *Ployodon spathula*; Lo, *Lepisosteus oculatus*.
