# Supplementary material for: Whole-genome Sequencing Reveals Autooctoploidy in Chinese Sturgeon and Its Evolutionary Trajectories
Source: Genomics Proteomics Bioinformatics. 2023 Dec 13;22(1):qzad002. doi: 10.1093/gpbjnl/qzad002 (PMC11425059; doi:10.1093/gpbjnl/qzad002)
Supplement: qzad002_Supplementary_Data [file qzad002_supplementary_data.zip › Table S13-by JieLiu by Chi-wbz.docx]

**Table S13 Statistics of gene family analysis based on** **OrthoFinder**

| **Type** | **Number** |
| --- | --- |
| Number of species | 13 |
| Number of genes | 356,603 |
| Number of genes in orthogroups | 342,794 |
| Number of unassigned genes | 13,809 |
| Percentage of genes in orthogroups | 96.1 |
| Percentage of unassigned genes | 3.9 |
| Number of orthogroups | 21,410 |
| Number of species-specific orthogroups | 2777 |
| Number of genes in species-specific orthogroups | 13,555 |
| Percentage of genes in species-specific orthogroups | 3.8 |
| Mean orthogroup size | 16 |
| Median orthogroup size | 15 |
| G50 (assigned genes) | 20 |
| G50 (all genes) | 19 |
| O50 (assigned genes) | 5037 |
| O50 (all genes) | 5396 |
| Number of orthogroups with all species present | 6757 |
| Number of single-copy orthogroups | 150 |
