# Supplementary material for: Whole-genome Sequencing Reveals Autooctoploidy in Chinese Sturgeon and Its Evolutionary Trajectories
Source: Genomics Proteomics Bioinformatics. 2023 Dec 13;22(1):qzad002. doi: 10.1093/gpbjnl/qzad002 (PMC11425059; doi:10.1093/gpbjnl/qzad002)
Supplement: qzad002_Supplementary_Data [file qzad002_supplementary_data.zip › Table S14-by JieLiu by Chi-wbz.docx]

**Table S14 Distribution of tetra-nucleotide repeats in the screening process**

| **Type** | **All tetra-nucleotide repeats** | **Agarose gel electrophoresis** | **polyacrylamide gel electrophoresis** | **Fluorescent genotyping** | **4*n*** | **8*n*** |
| --- | --- | --- | --- | --- | --- | --- |
| **Total** | 3303 | 1525 | 982 | 285 | 23 |  |
| **Available** | 1525 | 982 | 285 | 47 | 16 | 25 |
