# Supplementary material for: Whole-genome Sequencing Reveals Autooctoploidy in Chinese Sturgeon and Its Evolutionary Trajectories
Source: Genomics Proteomics Bioinformatics. 2023 Dec 13;22(1):qzad002. doi: 10.1093/gpbjnl/qzad002 (PMC11425059; doi:10.1093/gpbjnl/qzad002)
Supplement: qzad002_Supplementary_Data [file qzad002_supplementary_data.zip › Table S15-by JieLiu.docx]

**Table S15 Comparison of SNPs and heterozygosity**

| **Species** | **Number of SNPs** | **Genome size** | **Heterozygosity** |
| --- | --- | --- | --- |
| *Acipenser sisnensis* | 22,324,005 | 1,995,374,126 | 1.12% |
| *Acipenser ruthenus* | 9,826,321 | 1,830,501,248 | 0.54% |

*Note*: SNPs, single nucleotide polymorphisms.
