# Supplementary material for: Whole-genome Sequencing Reveals Autooctoploidy in Chinese Sturgeon and Its Evolutionary Trajectories
Source: Genomics Proteomics Bioinformatics. 2023 Dec 13;22(1):qzad002. doi: 10.1093/gpbjnl/qzad002 (PMC11425059; doi:10.1093/gpbjnl/qzad002)
Supplement: qzad002_Supplementary_Data [file qzad002_supplementary_data.zip › Table S16-by JieLiu-wbz.docx]

**Table S16 Homoeologous block with collinearity for specific repeats cluster**

|  | **Chr_S1** | **Start_S1** | **End_S1** | **Chr_S2** | **Start_S2** | **End_S2** | **Length_S1** | **Length_S2** | **Orientation** |
| --- | --- | --- | --- | --- | --- | --- | --- | --- | --- |
| Pair_block1 | Chr01 | 1,419,895 | 21,926,870 | Chr02 | 80,736,214 | 99,644,135 | 20,506,975 | 18,907,921 | + |
| Pair_block2 | Chr01 | 33,824,029 | 76,278,202 | Chr02 | 80,315,433 | 39,611,299 | 42,454,173 | 40,704,134 | - |
| Pair_block3 | Chr03 | 3,631,635 | 16,013,866 | Chr04 | 105,356,645 | 118,182,366 | 12,382,231 | 12,825,721 | + |
| Pair_block4 | Chr03 | 26,028,856 | 41,137,682 | Chr04 | 30,920,604 | 16,216,966 | 15,108,826 | 14,703,638 | - |
| Pair_block5 | Chr03 | 63,661,724 | 84,502,135 | Chr04 | 77,090,706 | 93,380,380 | 20,840,411 | 16,289,674 | + |
| Pair_block6 | Chr03 | 84,784,644 | 1.04E+08 | Chr04 | 32,532,931 | 53,556,948 | 19,389,826 | 21,024,017 | + |
| Pair_block7 | Chr05 | 7,056,132 | 35,147,168 | Chr06 | 19,050,301 | 47,758,394 | 28,091,036 | 28,708,093 | + |
| Pair_block8 | Chr05 | 43,074,499 | 58,183,191 | Chr06 | 54,347,797 | 69,566,717 | 15,108,692 | 15,218,920 | + |
| Pair_block9 | Chr05 | 58,811,613 | 76,308,321 | Chr06 | 71,210,576 | 85,335,017 | 17,496,708 | 14,124,441 | + |
| Pair_block10 | Chr05 | 76,384,583 | 91,922,197 | Chr06 | 18727,222 | 7,813,375 | 15,537,614 | 10,913,847 | − |
