# Supplementary material for: Whole-genome Sequencing Reveals Autooctoploidy in Chinese Sturgeon and Its Evolutionary Trajectories
Source: Genomics Proteomics Bioinformatics. 2023 Dec 13;22(1):qzad002. doi: 10.1093/gpbjnl/qzad002 (PMC11425059; doi:10.1093/gpbjnl/qzad002)
Supplement: qzad002_Supplementary_Data [file qzad002_supplementary_data.zip › Table S18-by JieLiu by Chi-wbz.docx]

**Table S18 Statistics of topological structures based on LORe and AORe**

| **Number of topologies** | **Rank in all structures** | **Number of syntenic genes** | **Proportion** |
| --- | --- | --- | --- |
| Total gene number | - | 1438 | - |
| (P(SR))-(P(SR)) | 1 | 451 | 31.36% |
| PP-((SR)-(SR)) | 2 | 235 | 16.34% |
| P((P-(SR))-(SR)) | 3 | 118 | 8.21.% |
| ((PP)-(SR))-(SR) | 4 | 73 | 5.08% |
| PP-((SS)-(RR)) | 5 | 50 | 3.48% |
| Other topological sutrctures | 6 | 511 | 35.53% |

*Note*: P, *Polyodon spathula*; S, *Acipenser sinensis*; R, *Acipenser ruthenus*; LORe, lineage-specific ohnologue resolution; AORe, ancestral ohnologue resolution.
