# Supplementary material for: Whole-genome Sequencing Reveals Autooctoploidy in Chinese Sturgeon and Its Evolutionary Trajectories
Source: Genomics Proteomics Bioinformatics. 2023 Dec 13;22(1):qzad002. doi: 10.1093/gpbjnl/qzad002 (PMC11425059; doi:10.1093/gpbjnl/qzad002)
Supplement: qzad002_Supplementary_Data [file qzad002_supplementary_data.zip › Figure S8.pdf]

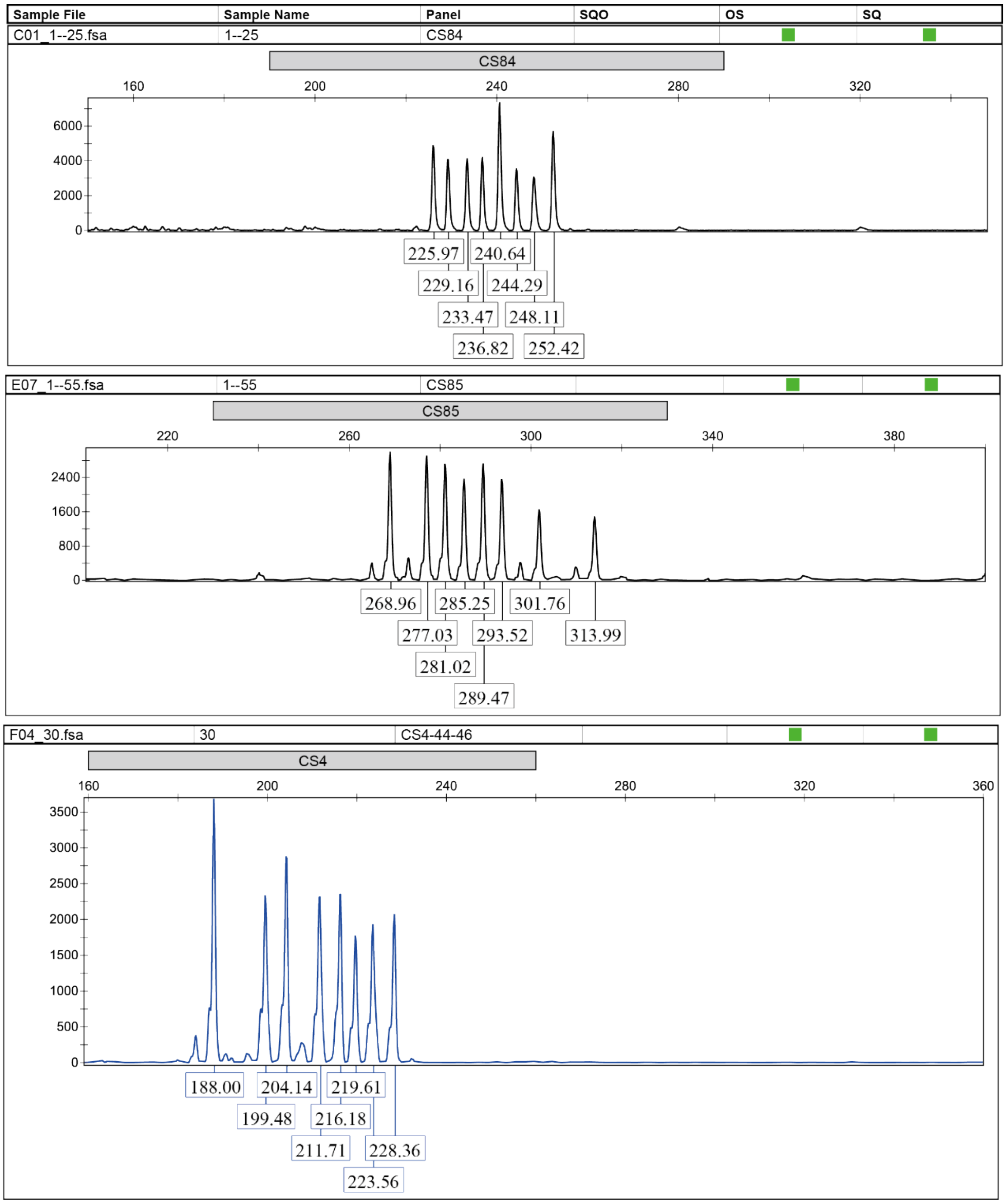
Sample File
Sample Name
Panel
SQO
OS
SQ
C01_1--25.fsa
1--25
CS84
CS84
160
200
240
280
320
6000
4000
2000
0
225.97
240.64
229.16
244.29
233.47
248.11
236.82
252.42
E07_1--55.fsa
1--55
CS85
CS85
220
260
300
340
380
2400
1600
800
0
268.96
285.25
301.76
277.03
293.52
313.99
281.02
289.47
F04_30.fsa
30
CS4-44-46
CS4
160
200
240
280
320
360
3500
3000
2500
2000
1500
1000
500
0
188.00
204.14
219.61
199.48
216.18
211.71
228.36
223.56
