# Supplementary material for: Whole-genome Sequencing Reveals Autooctoploidy in Chinese Sturgeon and Its Evolutionary Trajectories
Source: Genomics Proteomics Bioinformatics. 2023 Dec 13;22(1):qzad002. doi: 10.1093/gpbjnl/qzad002 (PMC11425059; doi:10.1093/gpbjnl/qzad002)
Supplement: qzad002_Supplementary_Data [file qzad002_supplementary_data.zip › Figure S3.pdf]

a

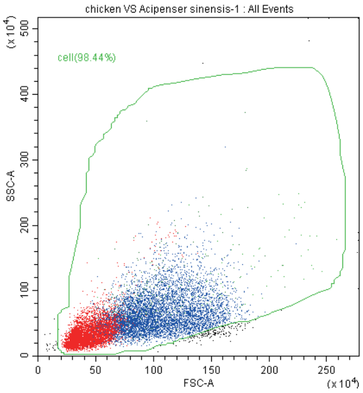

b

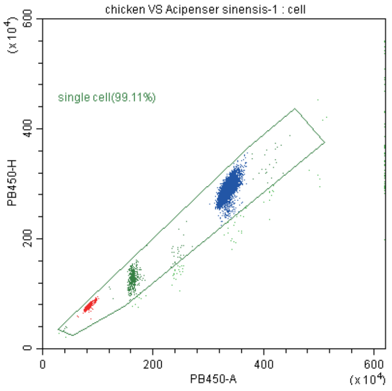

c

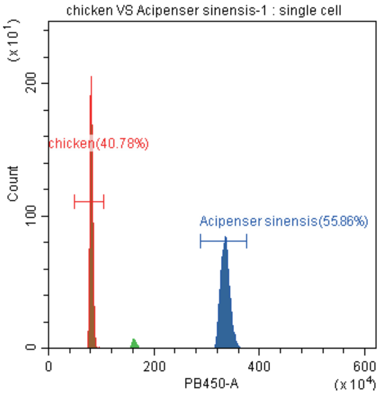

d

Tube Name: chicken VS Acipenser sinensis-1

Sample ID:

Population	Events	% Total	% Parent	CV PB450-A	Median PB450-A	Mean PB450-A
All Events	12880	100.00%	100.00%	58.67%	3242204.3	2296296.5
cell	12679	98.44%	98.44%	55.50%	3239217.8	2278178.0
single cell	12566	97.56%	99.11%	54.80%	3237777.5	2257395.5
chicken	5124	39.78%	40.78%	3.23%	809030.2	812057.1
Acipenser sinensis	7019	54.50%	55.86%	2.53%	3335678.5	3339971.0
