# Supplementary figures and images for: Whole-genome Sequencing Reveals Autooctoploidy in Chinese Sturgeon and Its Evolutionary Trajectories
Source: Genomics Proteomics Bioinformatics. 2023 Dec 13;22(1):qzad002. doi: 10.1093/gpbjnl/qzad002 (PMC11425059; doi:10.1093/gpbjnl/qzad002)

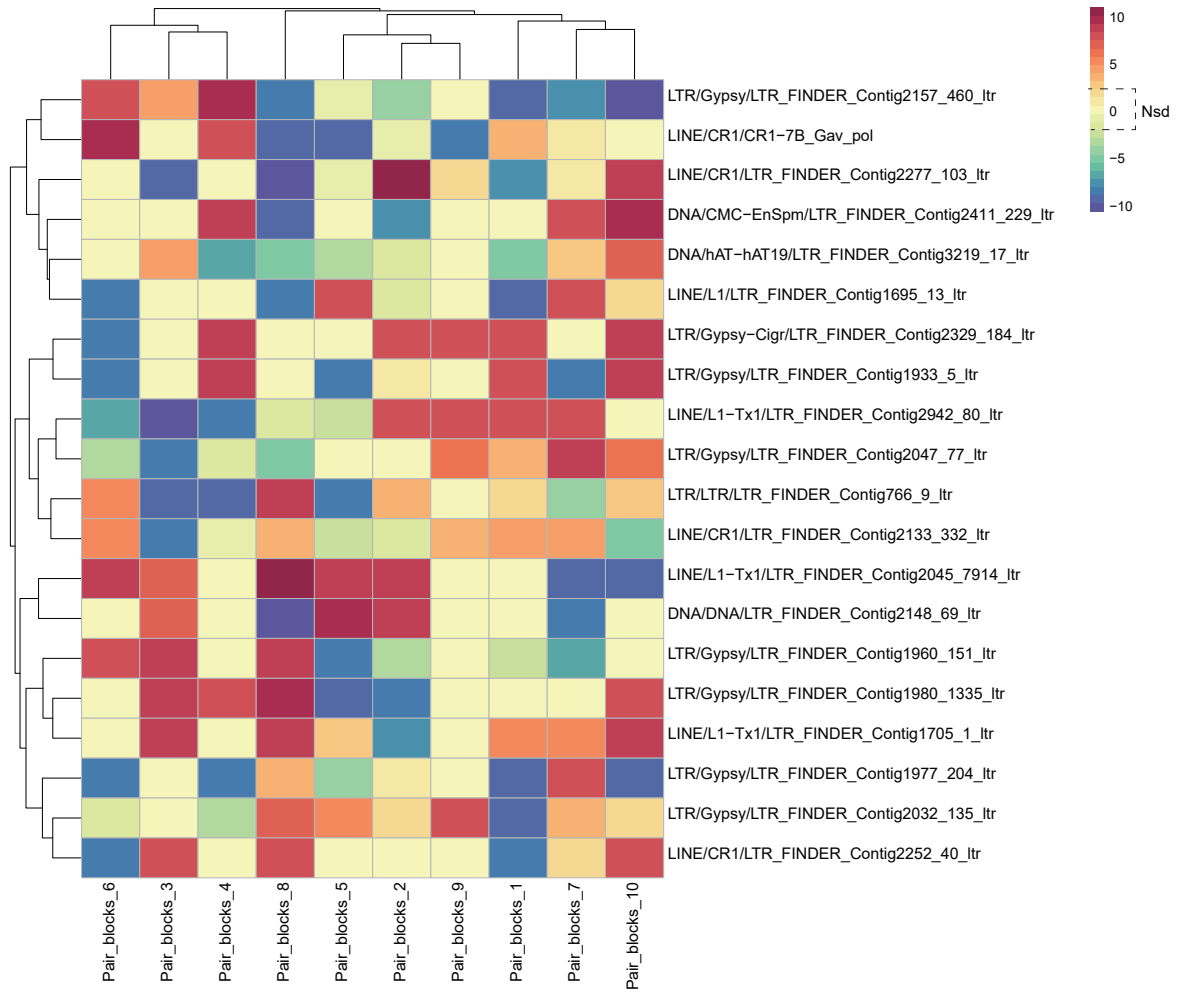

Supplement: qzad002_Supplementary_Data [file qzad002_supplementary_data.zip › Figure S9.pdf]

*Acipenser sinensis*

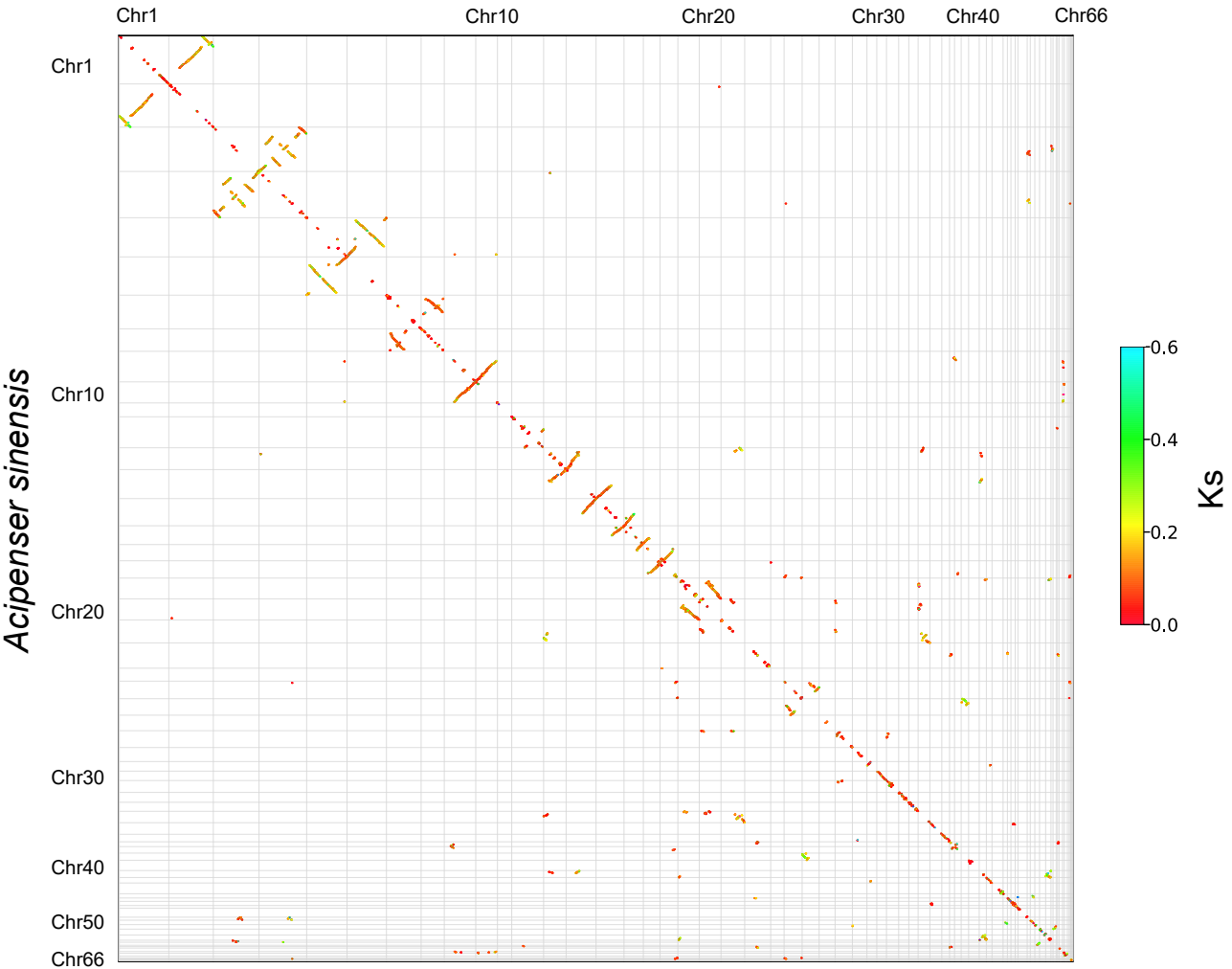

Supplement: qzad002_Supplementary_Data [file qzad002_supplementary_data.zip › Figure S10.pdf]

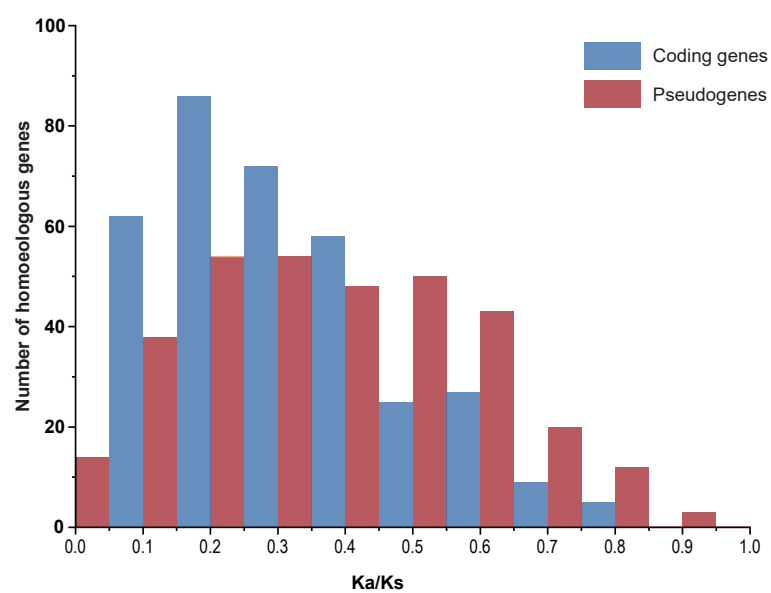

Supplement: qzad002_Supplementary_Data [file qzad002_supplementary_data.zip › Figure S11.pdf]

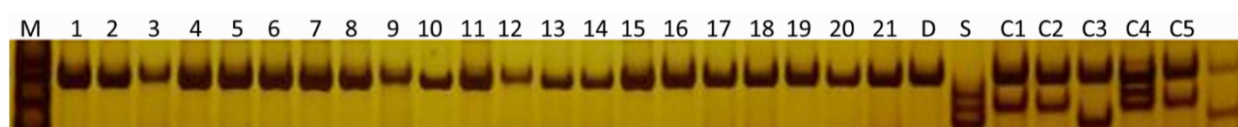

Supplement: qzad002_Supplementary_Data [file qzad002_supplementary_data.zip › Figure S13.pdf]

A

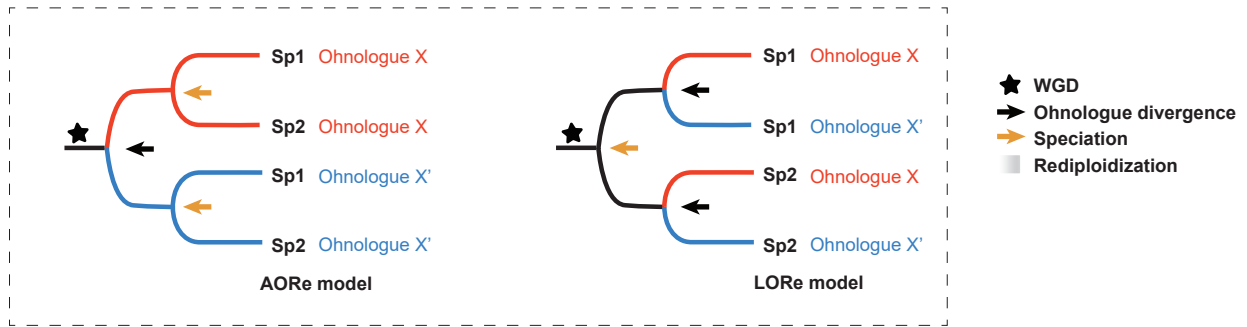

B

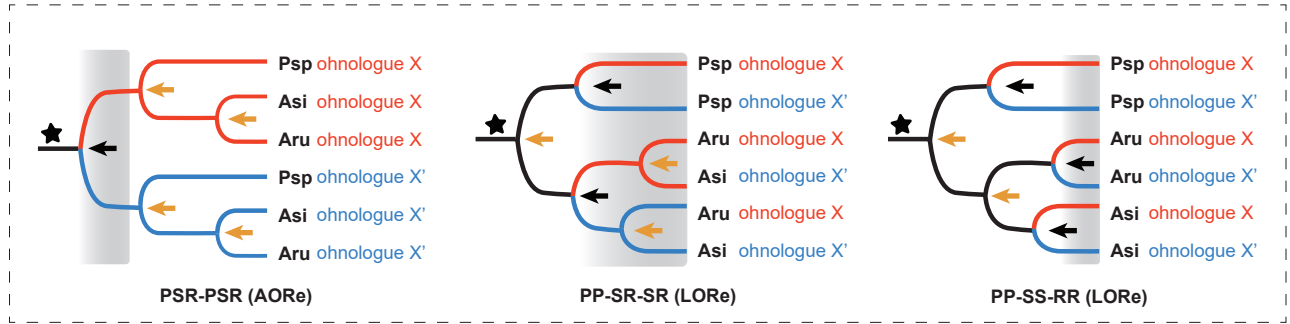

Supplement: qzad002_Supplementary_Data [file qzad002_supplementary_data.zip › Figure S1.pdf]

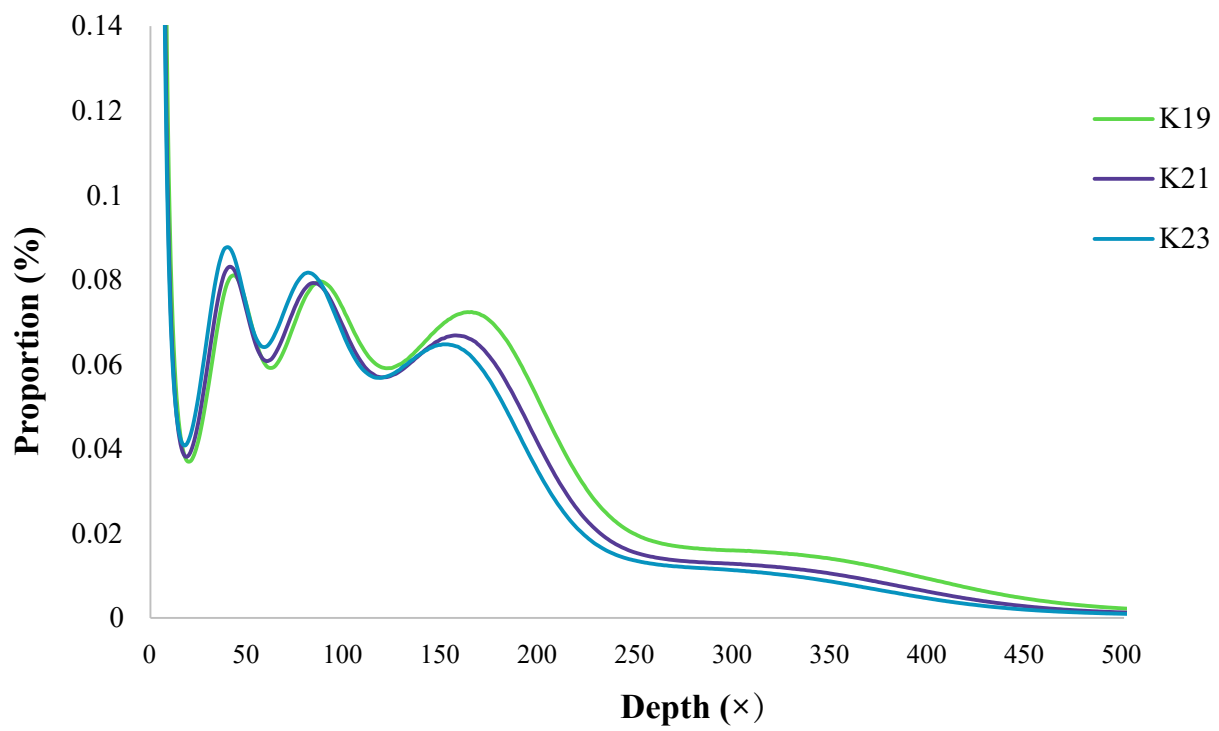

Supplement: qzad002_Supplementary_Data [file qzad002_supplementary_data.zip › Figure S2 .pdf]

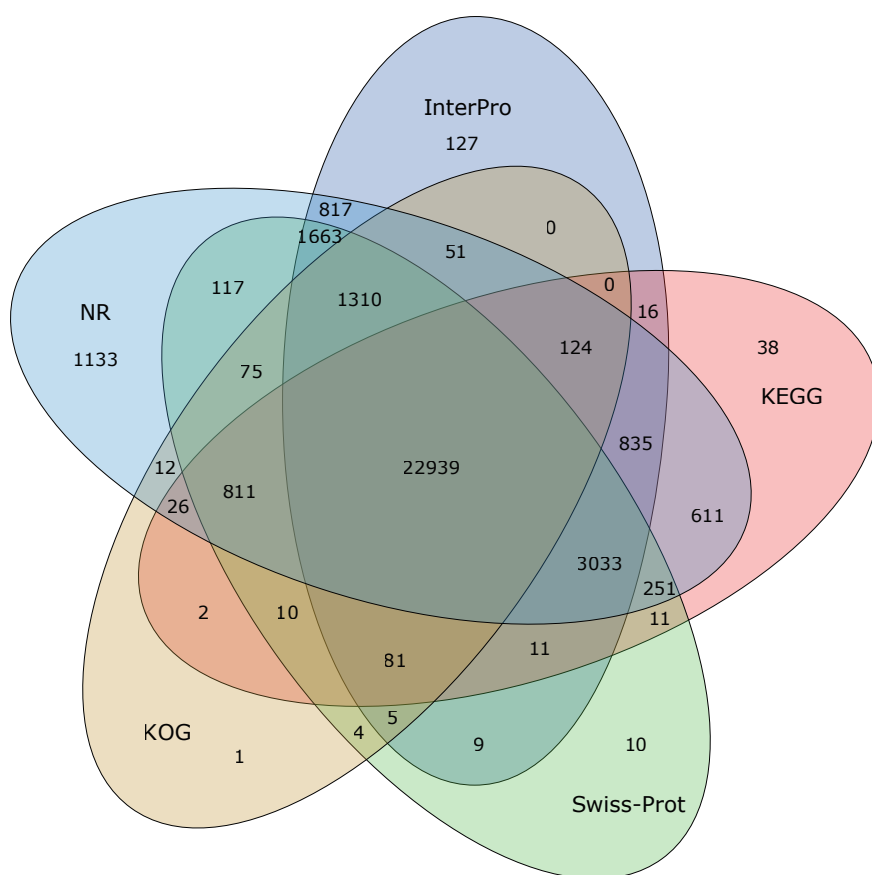

Supplement: qzad002_Supplementary_Data [file qzad002_supplementary_data.zip › Figure S4-Au111623.pdf]

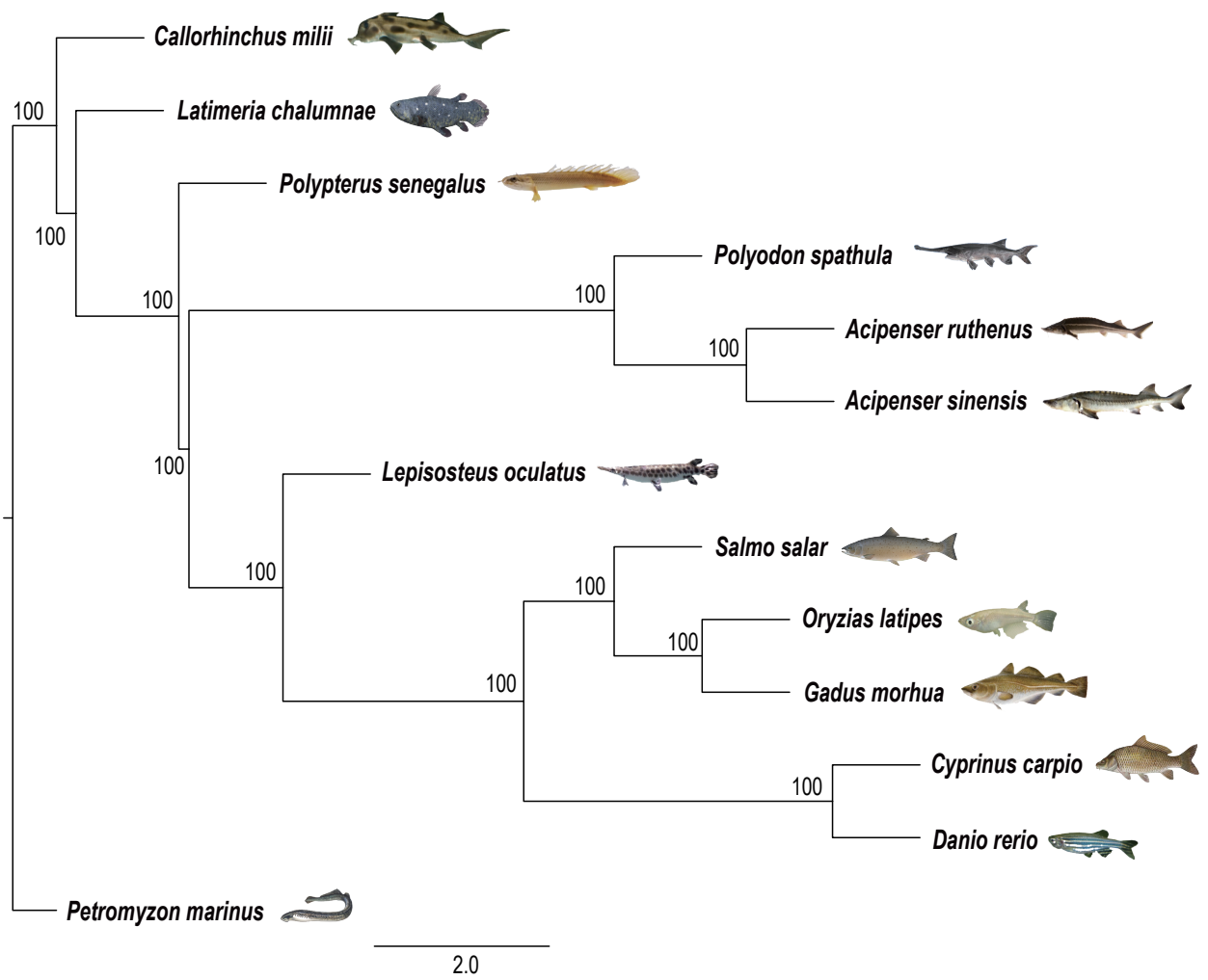

Supplement: qzad002_Supplementary_Data [file qzad002_supplementary_data.zip › Figure S5.pdf]

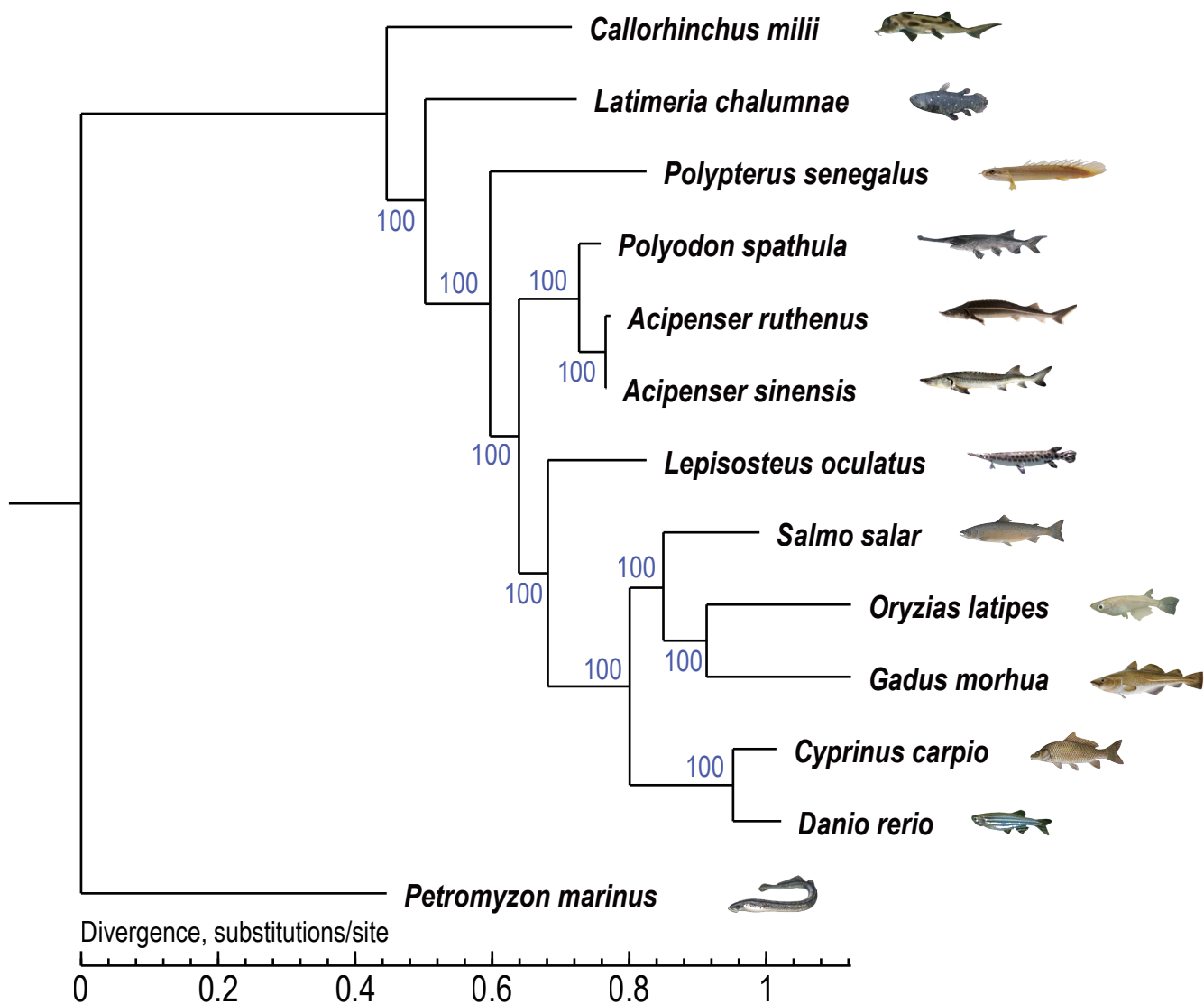

Supplement: qzad002_Supplementary_Data [file qzad002_supplementary_data.zip › Figure S6.pdf]

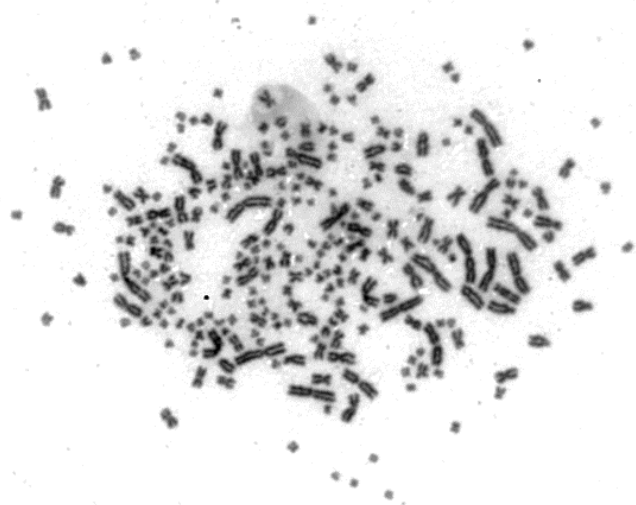

| 1111 | 1112 | 1113 | 1114 | 1115 | 1116 |
|------|------|------|------|------|------|
| 1117 | 1118 | 1119 | 1120 | 1121 | 1122 |
| 1123 | 1124 | 1125 | 1126 | 1127 | 1128 |
| 1129 | 1130 | 1131 | 1132 | 1133 | 1134 |
| 1135 | 1136 | 1137 | 1138 | 1139 | 1140 |
| 1141 | 1142 | 1143 | 1144 | 1145 | 1146 |
| 1147 | 1148 | 1149 | 1150 | 1151 | 1152 |
| 1153 | 1154 | 1155 | 1156 | 1157 | 1158 |
| 1159 | 1160 | 1161 | 1162 | 1163 | 1164 |
| 1165 | 1166 | 1167 | 1168 | 1169 | 1170 |

Supplement: qzad002_Supplementary_Data [file qzad002_supplementary_data.zip › Figure S7.pdf]
